# Validating Low‐Dose Iohexol as a Marker for Glomerular Filtration Rate by In Vitro and In Vivo Studies

**DOI:** 10.1111/cts.70141

**Published:** 2025-02-03

**Authors:** Qian Dong, Zhendong Chen, Jana Boland, Charalambos Dokos, Yohannes Hagos, Annett Kühne, Max Taubert, Dirk Gründemann, Uwe Fuhr

**Affiliations:** ^1^ Department of Pharmacology, Center for Pharmacology Faculty of Medicine and University Hospital Cologne, University of Cologne Cologne Germany; ^2^ PortaCellTec Biosciences GmbH Göttingen Germany

**Keywords:** clinical trial, glomerular filtration rate, Iohexol, transporter‐mediated drug–drug interactions

## Abstract

Clearance of an intravenous iohexol dose of 3235 mg is used to assess glomerular filtration rate (GFR), although systematic assessment of its pharmacokinetic (PK) properties is incomplete. The objectives of the present investigations were (i) to assess potential interactions of iohexol with important drug transporters, and (ii) whether a 259 mg dose could replace the current standard dose. In vitro, we evaluated whether iohexol inhibits or is transported by renal transporters (hOAT1/3, hOCT2, and hMATE1/2K) or other transporters (hOATP1B1/3, hOCT1, and hMDR1) using cell‐based and vesicle‐based systems. In vivo, we conducted a clinical trial with 12 volunteers with the administration of single intravenous doses of 3235 mg (“reference”) and 259 mg (“test”) using a changeover design. Plasma and urine samples were collected up to 24 h postdose. We assessed the dose linearity of iohexol pharmacokinetics using the standard bioequivalence approach and conducted a population PK analysis to characterize its profile. Our in vitro findings indicate that iohexol is neither a substrate nor a significant inhibitor of the transporters, suggesting it is unlikely to participate in transporter‐mediated drug–drug interactions in vivo. In the clinical trial, the test/reference ratio for plasma clearance, calculated as dose divided by the area under the plasma concentration–time curve, was 1.01 (90% confidence interval 0.968–1.05), confirming dose linearity. Population PK analysis further supported these results, showing no significant effect of dose on renal clearance and negligible nonrenal clearance of iohexol. Low‐dose iohexol is a suitable marker for precise GFR measurement, even when coadministered with other drugs.

**Summary**

- What is the current knowledge on the topic?
  ○ Estimating glomerular filtration rate (GFR) to assess kidney function is a key aspect of medical practice. While serum creatinine concentration is typically used for this purpose, results are error‐prone. Iohexol clearance of a standard 3235 mg intravenous bolus dose is more reliable in estimating GFR, but it is rarely used, partly due to limited research.
- What question did this study address?
  ○ This study investigated whether iohexol interacts with key drug transporters using in vitro methods and whether the iohexol dose to estimate GFR could be reduced to 259 mg in a clinical trial with healthy volunteers.
- What does this study add to our knowledge?
  ○ Iohexol is neither a substrate nor a significant inhibitor for transporters, suggesting that it is unlikely to interfere with other medications, and our clinical trial provided equivalent clearance values for both iohexol doses. Thus, a 259 mg iohexol bolus dose enables accurate GFR measurement, even when coadministered with other drugs.
- How might this change clinical pharmacology or translational science?
  ○ Clinically, the low iohexol dose enables accurate GFR measurement even in patients with fluctuating renal function and those with significant differences in non‐GFR determinants of creatinine clearance compared to the population used to develop the creatinine‐based GFR estimation equations. For the evaluation of renal transporter activity in clinical studies, the low iohexol dose fulfills the requirements for integration into a probe drug cocktail.

## 1 Introduction

Reliable quantification of renal function, specifically glomerular filtration rate (GFR) is essential for optimizing drug dosing in patients and evaluating drug pharmacokinetic (PK) properties in clinical research [1]. Serum creatinine concentrations and/or creatinine clearance are commonly used to this end [2]. However, renal elimination of creatinine is not only mediated by glomerular filtration but also by renal transporters, including human organic cation transporter (hOCT)2, 2 forms of human multidrug and toxin extrusion proteins (hMATE1 and hMATE2K), and human organic anion transporter (hOAT)2 [3]. Therefore, creatinine‐based GFR estimations may be biased and influenced by transporter‐mediated drug–drug interactions (TDDIs) [1, 3]. Cystatin C is less affected by renal tubular processes compared to creatinine, potentially making it a more reliable GFR marker [4]. However, both markers are affected by non‐GFR factors: serum creatinine concentrations are influenced by muscle mass, physical activity, and diet [5], while cystatin C is impacted by inflammation, metabolic disorders, and steroid use [6].

Alternatively, iohexol plasma clearance following a single dose (typically 3235 mg of iohexol) has become a robust GFR quantification method owing to its favorable PK properties [1]. Unlike endogenous filtration markers, iohexol‐based assessments are unaffected by variations in body composition or disease [2]. It is used in clinical settings, where precise GFR assessment is required, such as in patients with fluctuating kidney function [7, 8], and in clinical trials evaluating the role of GFR in drug pharmacokinetics [1, 9]. Iodinated contrast media (ICM), including iohexol, are generally well tolerated; however, adverse drug reactions (ADRs) occur in up to 3% of cases, with acute and prolonged effects [10]. Acute ADRs, including allergic‐like and physiologic responses, are dose‐dependent [10, 11]. Contrast‐induced acute kidney injury (CI‐AKI) is the primary serious ADR associated with ICM, with a low (1%–2%) in patients with normal renal function but increases to 25% in those with chronic kidney disease (CKD) or other risk factors, such as comorbidities, aging, or nephrotoxic drugs [12]. The volume of ICM injected is a critical determinant of CI‐AKI risk, with the risk doubling for every additional 20 mL in CKD patients [13]. Given the dose dependency of ADRs, it is desirable to validate and use iohexol at the lowest possible dose for GFR assessment, particularly in patients with impaired renal function or those requiring repeated GFR monitoring.

While iohexol elimination in humans is considered to be mediated exclusively by glomerular filtration, there is limited evidence suggesting that iohexol may interact with membrane transporters [9, 14, 15, 16]. Minor inconsistencies have been observed when comparing iohexol clearance to that of inulin, which is regarded as an ideal GFR marker [9], though inulin is no longer preferred due to practical limitations [17]. Additionally, beyond glomerular filtration iohexol might be reabsorbed through a saturable mechanism in rats [14]. Moreover, iohexol downregulated the expression of OCT2 in both rat kidneys and HK‐2 cells [15]. It also exerted a mild inhibitory effect on P‐glycoprotein in human cancer cell lines [16]. The involvement of membrane transporters in iohexol pharmacokinetics may lead to nonlinearity in its pharmacokinetics, particularly at low concentrations. Similar to creatinine, this can make iohexol susceptible to TDDIs when coadministered with drugs that affect transporter activity [3]. Both nonlinearity and TDDIs with iohexol as a victim could cause discrepancies between iohexol clearance and GFR.

TDDIs with iohexol as a perpetrator may affect drug therapy in patients. Furthermore, it could also influence the pharmacokinetics of probe drugs to assess the activity of renal transporters when integrated into “cocktail” studies. Cocktail studies are established approaches to quantify the activity of transporters (and enzymes) in vivo by simultaneous administration of several drugs, each of which is a substrate of a specific enzyme or transporter of interest [9]. Enzyme or transporter activities are quantified based on PK parameters representative of specific transporter activity. Several such cocktails have been developed specifically to assess the activity of renal transporters [18, 19, 20, 21]. Renal clearance of a probe drug depends on the activity of the respective transporter(s) and glomerular filtration. Evaluating net renal secretion, a primary metric for quantifying renal transporter activity, therefore requires an accurate assessment of GFR, for which iohexol plasma clearance may be a valuable tool [9]. However, to incorporate iohexol into future transporter cocktail approaches, it is essential to ensure that iohexol is not involved in relevant TDDIs. Furthermore, to minimize potential TDDIs, reduce the risk of adverse effects associated with iohexol exposure [11, 13], and lower iohexol consumption, a reduction in the standard dose is desirable. However, PK information on low‐dose iohexol is limited [7, 22].

This study, therefore, comprised two parts: The first part focused on assessing potential TDDIs of iohexol, including in vitro characterization of iohexol as a potential substrate and/or inhibitor of major drug transporters [23, 24]. The second part was a clinical study in healthy volunteers to assess the dose linearity of iohexol pharmacokinetics.

## 2 Materials and Methods

### 2.1 Part 1: In Vitro Study

#### 2.1.1 Study Design

We characterized the inhibitory potential of iohexol on major drug transporters recommended by regulatory agencies [23, 24], which were previously assessed in a clinical transporter phenotyping cocktail study [18]. These transporters include hOAT1, hOAT3, hOCT1, hOCT2, hMATE1, hMATE2K, human organic anion transporter polypeptides (hOATP)1B1, hOATP1B3, and human multidrug resistance protein (hMDR)1. Additionally, we investigated whether iohexol is a substrate for any of the renal transporters among these, including hOAT1/3, hOCT1/2, and hMATE1/2K. Cell‐based uptake assays were used to assess the activities of hOAT1/3, hOCT1/2, hMATE1/2K, and hOATP1B1/1B3. Inside‐out membrane vesicle uptake assays were used to evaluate the activity of hMDR1.

For substrate assessments, iohexol was incubated with stably transfected HEK‐293 cells expressing one of the following transporters: hOAT1, hOAT3, hOCT1, hOCT2, hMATE1, or hMATE2K, as well as with control cells lacking transporter expression. Stably transfected cell lines containing pEBTetD plasmids [25] with wild‐type human transporter cDNAs were generated as previously described [26]. Transporter expression was induced by adding 1 μg/mL doxycycline to the growth medium for at least 20 h [26]. Iohexol would be considered a substrate for these transporters if: (1) the ratio of iohexol uptake in cells expressing the transporter to that in control cells was ≥ 2, and (2) a known inhibitor of the transporter reduced iohexol uptake to ≤ 50% at concentrations ≥ 10 times its inhibition constant or half‐maximal inhibitory concentration [23, 24].

Inhibition experiments were conducted to investigate whether iohexol exhibits inhibitory effects on hOAT1/3, hOCT1/2, hMATE1/2K, hOATP1B1/3, and hMDR1 [18], potentially leading to TDDIs in vivo. To determine the maximal inhibitory potential of iohexol, intracellular accumulation of a standard substrate was measured with and without the clinically relevant highest concentrations of iohexol. Control inhibitors were tested in each experiment.

According to guidelines for assessing TDDIs after intravenous administration, the highest test concentration should be up to 50 times the unbound maximal plasma concentration (C_max_) [23, 24]. Given the minimal plasma protein binding of iohexol (1.5%) [27], we assumed an unbound fraction of 1 for the experiments. Typically, iohexol is administered as a 5 or 10 mL intravenous bolus (300 or 240 mg iodine/mL) for GFR measurement [27]. Based on prior clinical trials in patients aged ≥ 70 years with impaired kidney function (median GFR: 60.7 [interquartile range: 48.9–71.5] mL/min/1.73 m^2^), the C_max_ was considered to correspond to the initial plasma concentration following a 3235 mg iohexol injection, which was approximately 0.37 mM [28, 29]. Therefore, we selected a concentration range of 1–20 mM for the in vitro TDDI assessment to evaluate its maximum inhibitory potential.

In all subsequent in vitro studies, control experiments were conducted to validate the results. These included positive controls for substrates and inhibitors, as well as cells either without transporter expression or transfected with an empty vector, and control vesicles. Each condition, including cells or vesicles with the transporter and their respective controls, was analyzed in at least three replicates. For detailed information on materials, methods, and data analysis techniques used, refer to the Supporting Information. Additional details on the cell lines and culturing conditions are available in the related publication [26].

### 2.2 Part 2: In Vivo Study

#### 2.2.1 Study Design

The clinical trial was registered with the German Clinical Trials Register under the identification code DRKS00029908 and approved by the Ethics Committee of the Medical Faculty of the University of Cologne, Germany, on November 21, 2022 (number 22‐1347_1). The study adhered to Good Clinical Practice guidelines and the Declaration of Helsinki. All participants provided written informed consent before any study‐related procedures and were confirmed to be healthy through a standard screening examination (See also Supporting Information).

The trial had two separate objectives, that is, (i) to assess dose linearity of iohexol pharmacokinetics for a lower dose (reported here) and (ii) to improve the assessment of creatinine volume of distribution by oral administration of creatinine in beef meat (will be reported separately). Building on the PK findings from a 3235 mg iohexol injection [28, 29], we determined that the current quantification method is sufficiently sensitive to measure iohexol plasma concentrations corresponding to doses over 10 times lower, up to at least 20 h postadministration. Therefore, a 259 mg iohexol dose was selected for this study. The pertinent part of the trial had an open‐label, randomized, single‐dose, three‐period changeover design involving 12 volunteers. Each participant was randomly assigned to one of six sequences, receiving iohexol intravenously at different single doses on separate occasions: (1) 259 mg iohexol without beef ingestion (defined as “fasting,” “test”); (2) 3235 mg iohexol fasting (“reference”); and (3) 3235 mg iohexol with beef ingestion (not reported here). The washout interval between administrations was 7 to 14 days. Adverse events were surveyed until the completion of the study.

A total of 19 blood samples were drawn before iohexol administration and at 10, 20, 30, 45, 60, and 90 min, as well as at 2, 3, 4, 5, 6, 8, 10, 12, 14, 16, 20, and 24 h postdosing under fasting conditions. For urine, 11 samples were collected before dosing and at 0–2, 2–4, 4–6, 6–8, 8–10, 10–12, 12–14, 14–16, 16–20, and 20–24 h postdosing under fasting conditions. Iohexol concentrations in plasma and urine samples were quantified using validated high‐performance liquid chromatography coupled with tandem mass spectrometry, as detailed in the Supporting Information. Only data from the two fasting conditions were included in further analyses.

#### 2.2.2 Noncompartmental Analysis

The noncompartmental analysis (NCA) was conducted using PKanalix 2024R1 (Lixoft SAS, a Simulations Plus company, Paris, France) followed by comparing between doses using the bioequivalence module. To assess the potential nonlinear relationship between the dose and exposure of iohexol, plasma clearance (CL) estimates obtained from NCA were compared between doses using the standard bioequivalence approach [30]. Detailed information on the calculation of PK parameters via NCA and the statistical methods used is provided in the Supporting Information.

#### 2.2.3 Population Pharmacokinetic Analysis

A population PK model of iohexol was developed using the nonlinear mixed‐effects modeling program NONMEM version 7.5.0 (ICON plc, Dublin, Ireland), Perl speaks NONMEM (PsN) version 5.2.6 (Uppsala University, Uppsala, Sweden) [31], and Pirana version 3.0.0 (Certara, Princeton, New Jersey). R version 4.2.1 (R Foundation for Statistical Computing, Vienna, Austria) was used for data preparation, visualization, and statistical summaries.

The structural model was developed in a stepwise manner, as previously detailed [28]. After establishing a reasonable structural model, the impact of dose‐related effects was assessed as a categorical covariate on PK parameters through forward and backward selection processes. Model improvement was evaluated using the change in the objective function value (ΔOFV), with significance levels of 0.05 (ΔOFV ≤ −3.84) and 0.01 (ΔOFV ≤ −6.63). The stability and performance of the final model were evaluated graphically and statistically, using goodness‐of‐fit plots, nonparametric bootstrap analysis [32], and the visual predictive check (VPC) technique [33], as described earlier [28].

## 3 Results

### 3.1 Part 1: In Vitro Study

#### 3.1.1 Quality Assessment of In Vitro Systems

The in vitro systems for hOAT1/3, hOCT1/2, hMATE1/2K, hOATP1B1/3, and hMDR1 uptake assays demonstrated robust performance in this study. Reference substrate concentrations in cells or vesicles expressing the transporters were 3.3 to 250 times higher than in controls (Table S3). The median inhibitory effects of prototypical inhibitors on the uptake of their respective reference substrates across all transporters ranged from 49% to 99% (Table S3). These findings validate the functionality of the in vitro systems in our study.

#### 3.1.2 Substrate Assessments

**FIGURE 1:**
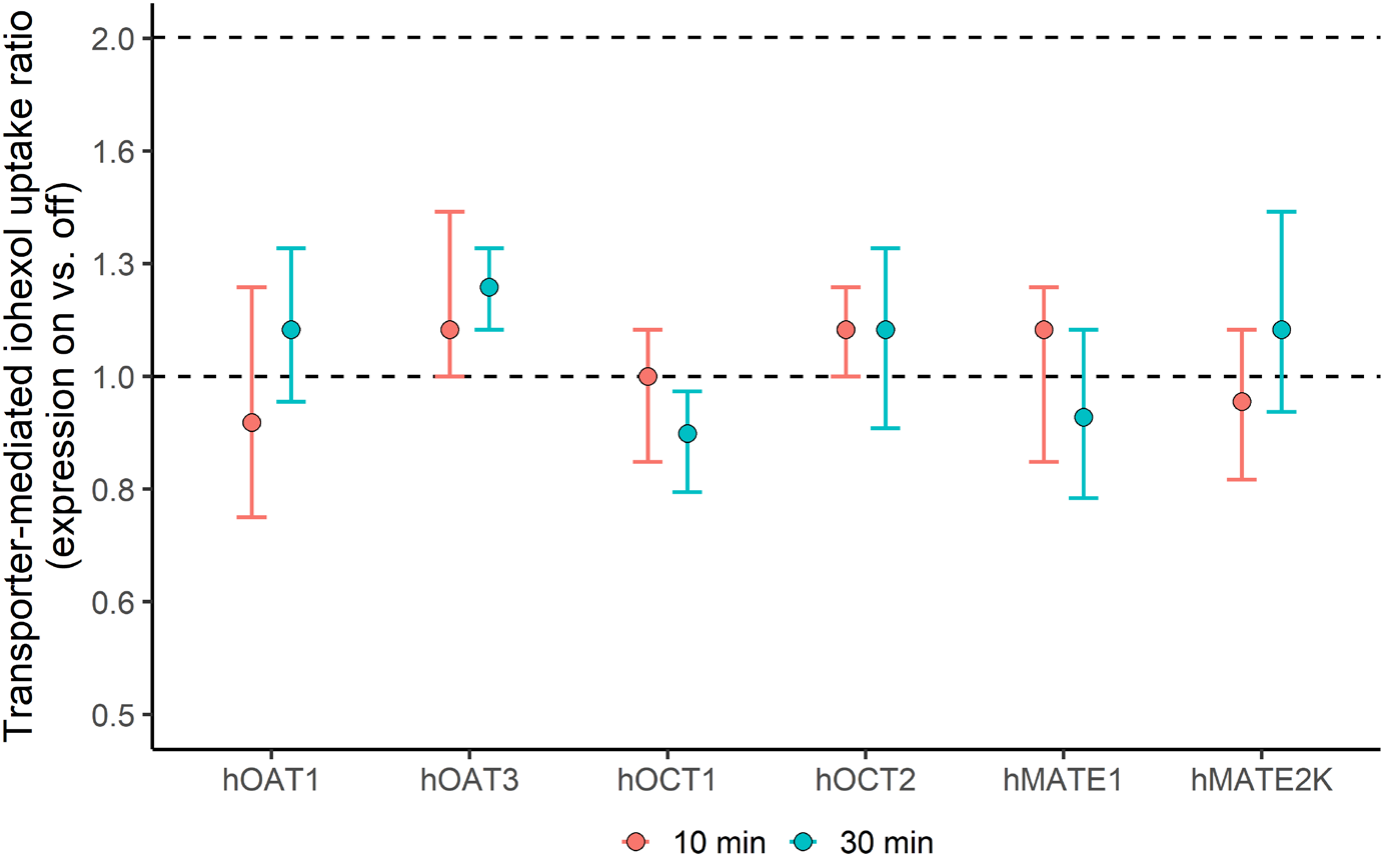
Ratio of iohexol uptake in transporter‐expressing cells vs. nonexpressing cells. Stably transfected 293 cells, either expressing (expression on; *n* = 3 or 4) or not expressing (expression off; *n* = 3 or 4) hOAT1, hOAT3, hOCT1, hOCT2, hMATE1, or hMATE2K, were incubated with 10 μM iohexol for 10 and 30 min, respectively. Each experiment was performed in triplicate, with each assay conducted on a separate day. In each assay, the ratio of iohexol uptake rates in transporter‐expressing cells (expression on) compared with nonexpressing cells (expression off) was calculated through element‐wise division within each experimental group. The dots and error bars represent the median values with 95% confidence intervals (CIs) of the log‐scaled ratios across three independent experiments.

Transporter‐expressing cells showed no significant increase in iohexol accumulation compared to control cells, with median values ranging from −0.67 to 1.5 pmol/mg protein after incubation with 10 μM iohexol for 10 and 30 min (Table S4, Figure S1). The median iohexol uptake ratio of transporter‐expressing cells to control cells varied between 0.89 and 1.2 across all transporters (Figure 1, Figure S1, Table S4).

#### 3.1.3 Inhibition Assays

**FIGURE 2:**
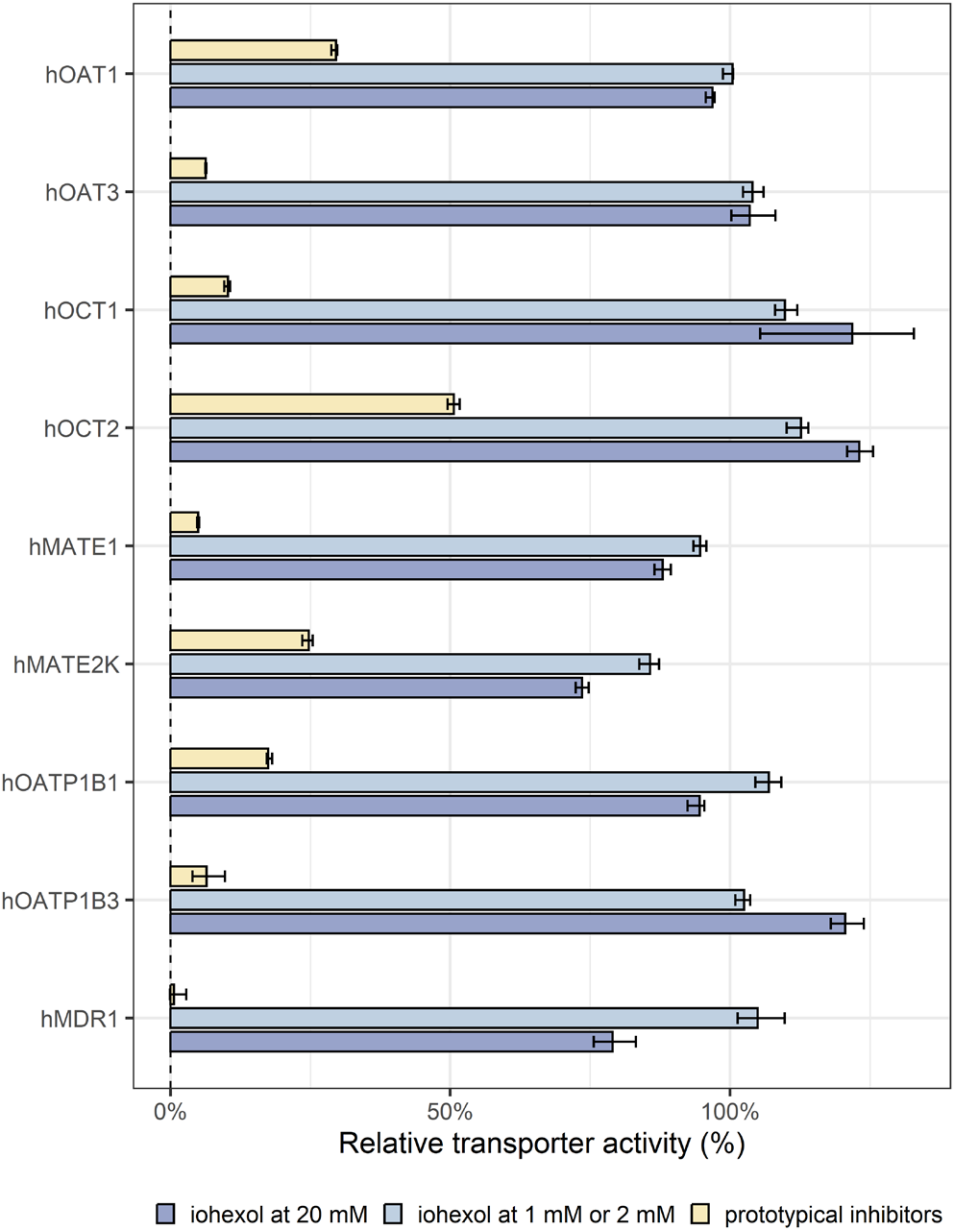
Inhibitory effects of prototypical inhibitors and iohexol on transporter‐mediated transport of standard substrates. Relative transporter activity was calculated as the ratio of activity in the presence of inhibitors or iohexol to the activity in their absence, using element‐wise division within each experiment. Transporter activity (net uptake rate) was derived by element‐wise subtraction of the probe substrate concentrations under “expression off” conditions from those under “expression on” conditions for each transporter and study group. Columns and error bars represent the median values and 95% CIs of relative transporter activity across all respective experiments.

As shown in Figure 2, iohexol exhibited no significant inhibitory effect on the transporter‐mediated uptake of standard substrates at concentrations of 1 mM and 2 mM. At 20 mM, iohexol reduced hMATE2K‐mediated MPP+ uptake and hMDR1‐mediated [^3^H]‐N‐methyl‐quinidine uptake by 26% and 21%, respectively, while no notable inhibitory effects were observed for hOCT1/2‐, hOAT1/3‐, hMATE1‐, or hOATP1B1/3‐mediated substrates uptake. Detailed results of the inhibition assays are shown in Figures S2 and S3 and Table S3.

### 3.2 Part 2 in Vivo Study

#### 3.2.1 Demographics and Dataset

Twelve healthy subjects (7 females) with a median body mass index of 24.4 kg/m^2^ (range: 21.2–28.9 kg/m^2^) and a median age of 34 years (range: 23–48 years) participated in the relevant part of the trial. Detailed demographic characteristics are presented in Table 1.

**TABLE 1:** Demographic summary of enrolled subjects (*n* = 12, 5 men, 7 women).

Characteristic	Median (range)
Age (years)	34 (23–48)
Weight (kg)	77.3 (59.1–95.8)
Height (cm)	178 (163–196)
BMI (kg/m^2^)	24.4 (21.2–28.9)
eGFR (mL/min/1.73 m^2^)	100 (83.6–134)

Abbreviations: BMI, body mass index; eGFR, estimated glomerular filtration rate, estimated using the 2021 Chronic Kidney Disease Epidemiology Collaboration (CKD‐EPI) creatinine–cystatin C equation [34], based on plasma creatinine and cystatin C concentrations from the screening examination.

**FIGURE 3:**
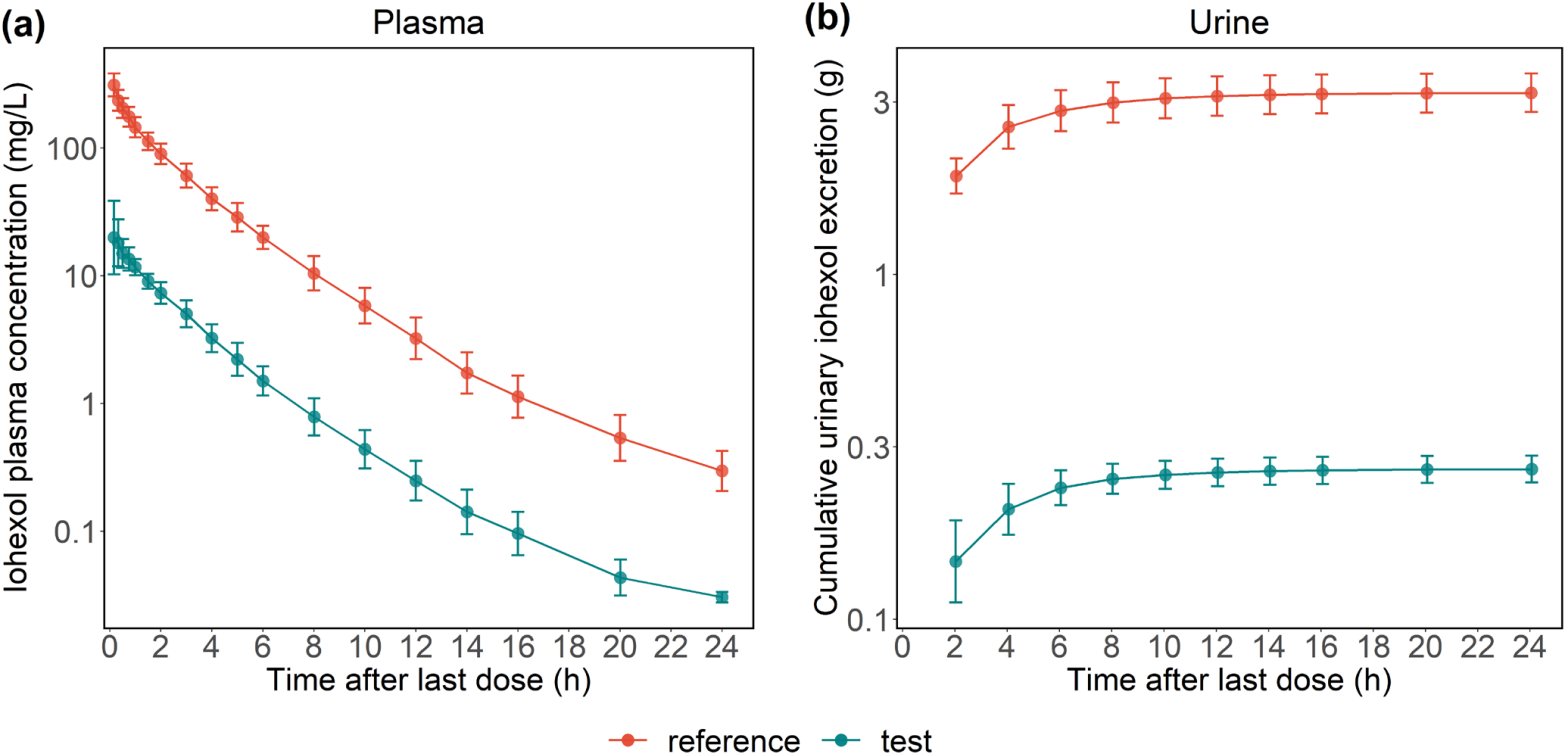
Semi‐logarithmic plots of (a) plasma concentration–time profiles of iohexol and (b) cumulative urinary excretion following single reference or test doses. Symbols and error bars represent geometric means and geometric standard deviations, respectively. Data from 12 subjects are included: 10 completed all sample collections, providing both plasma and urine data for the reference and test periods. One provided complete plasma data for both periods and urine data only in the reference period, while another provided complete plasma data for both periods and urine data only in the test period.

A total of 432 postdose plasma and 238 postdose urine samples were collected for PK analysis of iohexol. Of these, 9 samples (1.34%) had concentrations below the lower limit of quantification (LLOQ) and were excluded from the analyses (for details see Supporting Information). The plasma concentration–time profiles of iohexol, and its cumulative urinary excretion following single reference or test doses, are depicted in Figure 3.

#### 3.2.2 Noncompartmental Analysis

NCA was performed using plasma concentrations from 12 subjects and urine concentrations from 10 subjects who had no missing urine samples. The corresponding PK parameters of iohexol following test and reference doses are summarized in Table 2. The 90% confidence intervals (CIs) for the ratio of test to reference doses for CL, calculated as dose divided by the area under the plasma concentration‐time curve (AUC), and for urinary recovery were 1.01 (0.968–1.05) and 1.06 (0.960–1.17), respectively. These values fall within the standard bioequivalence range of 0.800–1.25, indicating that the AUC increased proportionally with the iohexol dose.

**TABLE 2:** Plasma and urine pharmacokinetic parameters of iohexol for test and reference doses by noncompartmental analysis (*n* = 12).

Samples (number of subjects)	Parameters (unit)	Test		Reference		T/R ratio (90% CIs)	CV_intra_ (%)
		Geomean (geoSD)	Geocv (%)	Geomean (geoSD)	Geocv (%)		
Plasma (12)	AUC_0‐t_ (mg·h/L)	46.5 (1.16)	14.8	598 (1.19)	17.4	—	—
	AUC_0‐∞_ (mg·h/L)	46.7 (1.16)	14.7	600 (1.19)	17.4	—	—
	t_1/2,λz_ (h)	3.32 (1.11)	10.2	4.14 (1.07)	6.97	—	—
	V_z_ (L)	26.1 (1.14)	13.2	32.4 (1.20)	18.6	—	—
	CL (L/h)	5.46 (1.15)	14.2	5.43 (1.19)	17.3	1.01 (0.968–1.05)	5.29
Urine (10^a^)	R_max_ (mg/h)	73.7 (1.27)	24.1	951 (1.12)	11.7	—	—
	Ae_0‐t_ (mg)	273 (1.09)	8.96	3364 (1.14)	13.0	—	—
	Recovery (%)	106 (1.13)	11.8	103 (1.13)	12.7	1.06 (0.960–1.17)	11.9

Abbreviations: Ae_0‐t_, cumulative urinary excretion of unchanged iohexol from administration to the last time point; AUC, area under the plasma concentration–time curve, AUC from time zero to the last time point and AUC from time zero extrapolated to infinity are represented by AUC_0‐t_ and AUC_0‐∞_, respectively; CI, confidence interval; CL, plasma clearance; CV_intra_, intraindividual coefficient of variation; geoCV, geometric coefficient of variation; Geomean, geometric mean; geoSD, geometric standard deviation; Recovery, percentage of the administered iohexol dose recovered in urine; R_max_, maximum observed excretion rate; T/R ratio, test‐to‐reference ratio; t_1/2,λz_, apparent terminal plasma elimination half‐life; V_z_, volume of distribution during pseudoequilibrium.

^a^ In two subjects, urine collection was not complete.

#### 3.2.3 Population Pharmacokinetic Analysis

##### 3.2.3.1 Model Building

The three‐compartment model with first‐order renal elimination best‐described plasma and urine data, reducing the OFV by 225 points compared to the two‐compartment model. Adding a nonrenal clearance did not improve the OFV (0.053‐point increase) and its estimate was negligible (0.001 L/h). The data confirmed that iohexol is exclusively eliminated by the kidneys through linear kinetics, without requiring more complex models such as nonlinear elimination.

The model was parameterized with central (V_1_) and peripheral (V_2_, V_3_) volumes of distribution, intercompartment clearances (Q_1_, Q_2_), and renal clearance (CL_R_) of iohexol. Interindividual variability was estimated for CL_R_, V_1_, V_2_, and V_3_ using an exponential model. Inter‐occasion variability for CL_R_, V_1_, and Q_2_ was linked to inter‐individual variability through an additive model. The residual variability in plasma and urine data was best described by proportional error models.

**FIGURE 4:**
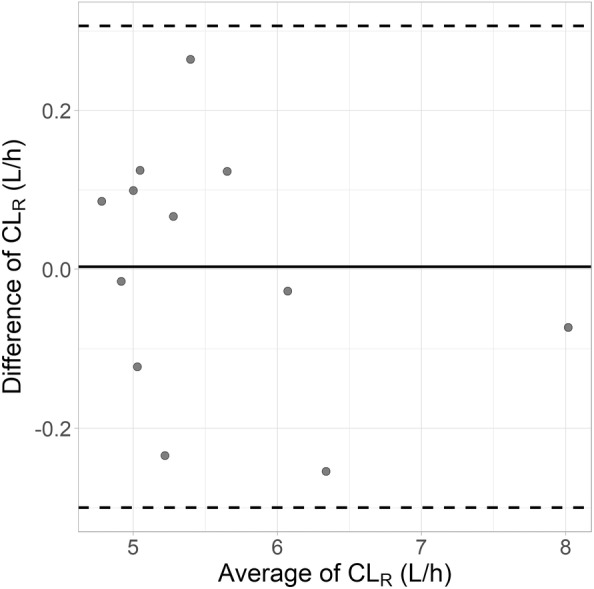
Bland–Altman plots comparing individual empirical Bayesian estimates (EBEs) of iohexol renal clearance following reference and test doses. The plot illustrates the difference between EBEs for renal clearance obtained after the test dose and those obtained after the reference dose. The mean bias of 0.00311 L/h is shown by the thin solid line, while the dashed lines denote the upper and lower limits of agreement.

Based on the final model, median (range) individual empirical Bayesian estimates (EBEs) were 5.25 (4.74–8.05) L/h for CL_R_ and 15.1 (11.5–18.8) L for the volume of distribution (sum of V_1_, V_2_, and V_3_). Bland–Altman plots indicated no significant differences in the individual EBEs of CL_R_ between the test and reference doses (Figure 4). Details of other PK parameters are presented in Table 3.

**TABLE 3:** Population pharmacokinetic parameters of iohexol and bootstrap results (*n* = 12).

Parameter (unit)	Point estimate	RSE%	Bootstrap median (95% CI)
Fixed effect			
CL_R_ (L/h)	5.50	4.22	5.50 (5.13–6.02)
V_1_ (L)	9.06	4.88	9.06 (8.18–10.1)
Q_1_ (L/h)	0.221	14.7	0.219 (0.164–0.313)
V_2_ (L)	1.56	7.74	1.57 (1.36–1.87)
Q_2_ (L/h)	5.84	18.5	5.76 (4.11–8.23)
V_3_ (L)	4.34	7.37	4.34 (3.81–4.99)
Interindividual variability (CV%)			
CL_R_	14.0	29.5	13.5 (5.05–20.1)
V_1_	16.6	24.0	15.5 (6.88–23.1)
V_2_	14.0	21.3	13.4 (7.81–20.7)
V_3_	15.5	18.8	13.7 (5.57–19.2)
Interoccasion variability (CV%)			
CL_R_	2.51	29.1	2.44 (0.900–3.87)
V_1_	6.23	39.2	6.06 (1.55–10.2)
Q_2_	29.2	50.0	28.7 (6.99–65.8)
Residual unexplained variability (CV%)			
Plasma	11.3	17.9	11.2 (8.33–14.7)
Urine	25.0	20.7	24.8 (14.7–36.8)

*Note:* CV% for interindividual and interoccasion variability computed as $\sqrt{\exp\left(\omega^{2}\right)-1}$, CV% for residual unexplained variability computed as $\sqrt{\exp\left(\sigma^{2}\right)-1}$.

Abbreviations: CI, confidence interval; CL_R_, renal clearance; CV%, coefficient of variation expressed as a percentage; Q_1_ and Q_2_, intercompartmental clearances; RSE%, relative standard error expressed as a percentage; V_1_, central volume of distribution; V_2_ and V_3_, peripheral volumes of distribution.

##### 3.2.3.2 Evaluating Dose‐Related Effects

Dose levels were tested as a categorical covariate in the analysis of PK parameters. No significant reduction in the OFV was observed, confirming that the PK parameters did not differ significantly across the different dose levels.

##### 3.2.3.3 Model Evaluation

The final model showed good agreement between predicted and observed data, though a few outliers were noted. For more details, refer to the Supporting Information.

## 4 Discussion

In this study, we investigated whether iohexol inhibits renal transporters (hOAT1/3, hOCT2, and hMATE1/2K), hepatic transporters (hOATP1B1/3), and transporters in various tissues (hOCT1, hMDR1) and whether it is a substrate for these renal transporters through laboratory‐based assays. Additionally, we assessed the PK characteristics of iohexol by comparing a low‐test dose to the usual reference dose in a clinical trial with healthy volunteers.

### 4.1 In Vitro Studies

In control experiments, the functional expression of each transporter was confirmed by the uptake of standard substrates. The use of specific inhibitors for each transporter effectively inhibited substrate uptake as expected, consistent with our previous study [26]. Thus, the results of these experiments should be reliable.

The potential interactions between iohexol and key drug transporters were evaluated through a series of in vitro experiments, which had not been systematically investigated previously. The results indicate that iohexol is neither a substrate nor a significant inhibitor for the investigated transporters. At a concentration of 20 mM, iohexol slightly inhibited hMATE2K and hMDR1 activities (< 30%). However, this concentration is far above the levels expected with the clinical dose (3235 mg) of iohexol used for GFR measurement or the reduced dose evaluated in this study. Thus, the mild inhibitory effect observed at this concentration is clinically irrelevant for using iohexol as a GFR probe. Similarly, Supawat et al. reported mild inhibitory effects of iohexol on P‐glycoprotein in human cancer cell lines. However, this inhibition was not statistically significant, raising doubt about the existence of this effect [16]. These in vitro findings align with clinical evidence [35, 36, 37, 38]. Studies have shown that iohexol clearance remains unaffected by OCT2 and MATE inhibitors, supporting the absence of significant interaction with these transporters [35, 36]. Furthermore, the low contribution of genetic polymorphisms to iohexol renal clearance variability suggests that genetically polymorphic transporters play a negligible role in its elimination [37]. Finally, iohexol does not induce metabolic drug–drug interactions, as it does not inhibit human Phase I or Phase II enzymes [38].

Our in vitro results, however, do not fully explain the minor discrepancies between iohexol‐based GFR measurements and the unavailable “gold standard” inulin‐based GFR [9, 39]. Furthermore, the discrepancy between our findings and those of Masereeuw et al., who proposed a saturable mechanism for iohexol elimination in the rat‐isolated perfused kidney, may arise from methodological limitations [14]. Masereeuw et al. observed an increase in the ratio of renal clearance to GFR (from 0.63 ± 0.06 to 1.02 ± 0.06, mean ± standard deviation) as perfusate concentrations increased from 5 μg/mL to 20 μg/mL, suggesting saturable reabsorption [14]. However, their use of cyanocobalamin for GFR measurement, relying on colorimetric assay may be less accurate at low concentrations [40], and increasing protein binding further undermines its reliability [41]. These limitations warrant caution in interpreting their conclusions. Similarly, the downregulation of OCT2 expression in rat kidneys and HK‐2 cells reported by Yang et al. also requires scrutiny [15]. Firstly, OCT2 expression is inconsistently observed in HK‐2 cells across studies [42], suggesting that any observed downregulation might not be specific to iohexol but could result from other factors or experimental conditions. Moreover, decreased OCT2 expression in contrast‐induced nephropathy rats may reflect nonspecific injury rather than a direct effect of iohexol [15]. Finally, relying on a single concentration of iohexol (6 mg/mL iodine) limits the robustness of the findings [15].

The variability in uptake and inhibition assays across experiments on different days, consistent with previous investigations [26], may result from differences in cell density and transfection age, which affect the number of active transporters in the plasma membrane. However, this variability does not compromise the pivotal results. Overall, our findings demonstrate that iohexol is neither a substrate nor a significant inhibitor of major drug transporters in vitro [23, 24].

### 4.2 Clinical Trial

We conducted a clinical trial to evaluate the feasibility of using a lower iohexol dose for GFR assessment by investigating the dose linearity of iohexol pharmacokinetics. In this trial, dose proportionality was assessed using two methods. First, the standard average bioequivalence approach was applied, utilizing noncompartmental PK evaluation. This method compares dose‐adjusted AUCs and employs well‐established criteria to confirm the absence of significant differences between doses without additional assumptions. Second, a population PK analysis was performed to gain a more detailed understanding of iohexol pharmacokinetics. The population PK employed a three‐compartment model that accurately described both plasma concentration profiles and urinary excretion of iohexol, consistent with prior findings in elderly individuals with impaired renal function based on plasma data alone [28]. Standard model evaluation and sensitivity analyses confirmed the stability and reliability of the final model. Iohexol PK parameters were unaffected by the dose, supporting the suitability of low‐dose iohexol (e.g., 259 mg) for GFR measurement.

Our results are consistent with previous research indicating iohexol plasma clearance as a reliable GFR comparable to inulin clearance [27, 43]. The iohexol clearance estimates agree with those reported for healthy adults, including the median renal clearance of 6.78 L/h/1.73 m^2^ (interquartile range: 6.36–7.50 L/h/1.73 m^2^) by Sterner et al. [39], and 7.32 L/h (95% CI: 7.08–7.68 L/h) by Olsson et al. [44]. The intraindividual coefficient of variation for iohexol plasma clearance in this study (5.29%) was within the reported range of 5.6%–11.4% [27]. Other PK parameters, such as the volume of distribution estimated using the population PK analysis approach, were consistent with the previous three‐compartment model estimate (median 15.09 L) [28] and comparable to the reported 0.27 L/kg in healthy volunteers [44]. Discrepancies between the volumes of distribution obtained from the population PK evaluation (representing the steady‐state volume) and the pseudoequilibrium volume (V_z_) obtained by NCA are attributed to ongoing distribution processes during the apparent terminal elimination phase in iohexol plasma concentration–time profiles [45]. This also applies to differences in t_1/2,λz_ and V_z_ between periods with different iohexol doses, which cannot be described properly by NCA.

Iohexol has many characteristics of an ideal GFR marker. Including minimal protein binding and exclusive elimination via glomerular filtration without tubular reabsorption or secretion [27], as comfirmed in this study. Using lower doses of iohexol to minimize potential toxicity may be of special interest for critically ill patients or those at risk of AKI, where frequent or even continuous monitoring of unstable GFR is crucial for understanding the impact of physiological and pathological changes on renal function. Continuous low‐dose iohexol infusion has been shown to accurately track GFR changes, although further validation with larger sample sizes is needed [7, 22]. By improving the sensitivity of the previously reported analytical method [46], we decreased the LLOQ to 25 ng/mL, successfully measuring 91.7% of plasma iohexol concentrations 20 h after a single bolus dose of 259 mg. This method enables a tenfold reduction in iohexol doses compared with Dixon et al.'s study [7, 22], extending GFR monitoring periods while keeping total doses within safe limits.

The absence of transporter‐mediated iohexol uptake in vitro, combined with the lack of nonrenal elimination pathways and the negligible impact of dose on iohexol pharmacokinetics in vivo, confirms that iohexol does not significantly interact with major drug transporters [23, 24]. This supports the conclusion that clinically relevant TDDIs with iohexol are highly unlikely. Therefore, iohexol meets the necessary criteria for inclusion as a GFR probe drug in our established transporter phenotyping cocktail [18], or in other respective cocktails [19, 20, 21]. Additionally, iohexol is not expected to interact with coadministered drugs during GFR measurements.

The study's main limitation is its exclusive focus on healthy volunteers and the use of dense sampling, which may not be directly applicable to patients. However, this sampling schedule is neither intended nor necessary for clinical practice, as established limited sampling strategies for reliable GFR assessment with iohexol are available [47], and can also be applied to low‐dose iohexol. In critically ill patients, variations in GFR and the volume of distribution may impact iohexol pharmacokinetics [48, 49]. While there is no reason to believe that deviations from dose linearity would differ between healthy volunteers and patients with renal impairment, further validation studies are recommended to strengthen confidence in its clinical use in these populations.

### 4.3 Overall Conclusion

Our results demonstrate that iohexol does not interact with major drug transporters and is eliminated exclusively by a nonsaturable renal elimination, and confirmed dose proportionality of iohexol pharmacokinetics in vivo. Based on these findings, a 259 mg dose of iohexol is suitable for precise GFR measurement in clinical settings and as part of a probe drug cocktail to enable the evaluation of renal transporter activity in clinical studies.

## Author Contributions

Q.D. and U.F. wrote the manuscript. U.F. and D.G. designed the research. Q.D., Z.C., J.B., C.D., Y.H., A.K., and U.F. performed the research. Q.D., Z.C., M.T., Y.H., and A.K. analyzed the data.

## Conflicts of Interest

The authors declare no conflicts of interest.

## Supporting information


Appendix S1.
