# Supplementary material for: Validating Low‐Dose Iohexol as a Marker for Glomerular Filtration Rate by In Vitro and In Vivo Studies
Source: Clin Transl Sci. 2025 Feb 3;18(2):e70141. doi: 10.1111/cts.70141 (PMC11788587; doi:10.1111/cts.70141)
Supplement: Supplementary file 1 — Appendix S1. [file CTS-18-e70141-s001.docx]

**Title:** Validating low-dose iohexol as a marker for glomerular filtration rate by *in vitro* and *in vivo* studies

**Author:** Qian Dong^1^, Zhendong Chen^1^, Jana Boland^1^, Charalambos Dokos^1^, Yohannes Hagos^2^, Annett Kühne^2^, Max Taubert^1^, Dirk Gründemann^1^, Uwe Fuhr^1^

**Institution:**

1. Department of Pharmacology, Center for Pharmacology, Faculty of Medicine and University Hospital Cologne, University of Cologne, Cologne, Germany.

2. PortaCellTec Biosciences GmbH, Göttingen, Germany.

**Authors' ORCID IDs:**

Qian Dong: 0000-0001-7201-7643

Zhendong Chen: 0000-0002-2035-635X

Jana Boland: 0009-0006-4660-5816

Charalampos Dokos: 0000-0002-7883-2558

Yohannes Hagos: 0009-0006-0207-0936

Annett Kühne: 0009-0000-7314-2761

Max Taubert: 0000-0001-8925-7782

Dirk Gründemann: 0000-0003-0914-0299

Uwe Fuhr: 0000-0001-7087-5871

**Corresponding author contact information:**

Qian Dong

Department of Pharmacology, Center for Pharmacology, Faculty of Medicine and University Hospital Cologne, University of Cologne, Gleueler Straße 24, Cologne 50931, Germany

Email: [qdong2@smail.uni-koeln.de](mailto:qdong2@smail.uni-koeln.de)

**Funding:**

No funding was received for this work. However, Qian Dong and Zhendong Chen received scholarships from the China Scholarship Council to support their PhD studies.

**Conflicts of Interest:**

The authors declared no competing interests for this work.

**Keywords:** Glomerular filtration rate, Iohexol, Transporter-mediated drug-drug interactions, Clinical trial

**SUPPLEMENTARY MATERIAL**

# MATERIALS

Iohexol, cimetidine, and para-aminohippuric acid (PAH) were purchased from Cayman Chemical (Ann Arbor, MI, USA), while Iohexol-d5 was purchased from Toronto Research Chemicals (Toronto, Ontario, Canada). Metformin, decynium-22, 1-methyl-4-phenylpyridinium (MPP+), Estrone 3-sulfate (E3S), Elacridar, cyclosporine A, and probenecid were acquired from Sigma-Aldrich (Darmstadt, Germany). [^3^H]Estrone sulfate, ammonium salt, [6,7-^3^H(N)] with a specific activity of 40 Ci/mmol was purchased from American Radiolabeled Chemicals (Saint Louis; MO, USA), [^3^H]BSP with a specific activity of 10.2 Ci/mmol was obtained from Hartmann Analytics (Braunschweig, Germany), and [^3^H]NMQ (N-methyl-quinidine) with a specific activity of 69 Ci/mmol was purchased from SOLVO (Hungary). All other chemicals were at least of analytical grade.

# METHODS

## Part 1. *In vitro* study

### Uptake assays for hOCT, hOAT, and hMATE transporters

Uptake assays were performed using stably transfected 293 cells, with or without the expression of one of the following transporters: hOAT1, hOAT3, hOCT1, hOCT2, hMATE1, or hMATE2K, as previously described.[^1^](#_ENREF_1)

For the substrate assessment, cells were incubated with 10 µM iohexol for 10 minutes and 30 minutes for each transporter. Inhibition assays were performed using specific standard substrates for each transporter: 10 µM para-aminohippuric acid (PAH) for hOAT1, 3 µM estrone-3-sulfate (E3S) for hOAT3, 50 µM metformin for hOCT1, 5 µM metformin for hOCT2, and 3 µM 1-methyl-4-phenylpyridinium (MPP+) for hMATE1 and hMATE2K. These substrates were tested either alone or in combination with iohexol at concentrations of 1 mM and 20 mM, respectively, or with specific positive control inhibitors: 50 µM probenecid for hOAT1, 100 µM probenecid for hOAT3, 10 µM decynium-22 for hOCT1, and 100 µM cimetidine for hOCT2, hMATE1, and hMATE2K. To correct for the increased osmolarity due to the addition of 20 mM iohexol, 10 mM NaCl was removed from the uptake buffer, resulting in a final composition of 115 mM NaCl, 25 mM HEPES-NaOH (pH 7.4), 5.6 mM (+) glucose, 4.8 mM KCl, 1.2 mM KH_2_PO_4_, 1.2 mM CaCl_2_, and 1.2 mM MgSO_4_. Incubation times were 1 minute for hOAT1, hOAT3, and hOCT2, 20 minutes for hOCT1, and 0.5 minutes for hMATE1 and hMATE2K. The uptake was terminated by washing the cells with the ice-cold uptake buffer. The cells were then lysed with 1 mL of methanol for at least 20 minutes and stored at -20 °C. For substrate assessment, the methanol used for cell lysis included 2 ng/mL of iohexol-d5 as an internal standard.

The concentrations of probe drugs in cell lysate samples from uptake experiments were determined using liquid chromatography coupled with tandem mass spectrometry (LC-MS/MS), as detailed in the “LC-MS/MS analysis” section. Protein content in the mass spectrometry samples was estimated from three paired dishes using the BCA (bicinchoninic acid) assay (Pierce; Thermo Fisher 23225, Life Technologies, Darmstadt, Germany) with bovine serum albumin as the standard, following the previously described method.[^1^](#_ENREF_1)

### Uptake assay for hOATP1B1 and hOATP1B3

Uptake assays were carried out with stably transfected 293 cells expressing hOATP1B1 or hOATP1B3, as well as cells containing an empty vector, using the method described by Hsin CH, *et al.*.[^1^](#_ENREF_1) For the inhibition assays, cells were incubated with the radiolabeled standard substrates specific to each transporter: 0.01 μM ^3^H-estrone sulfate for hOATP1B1, and 0.02 μM ^3^H-bromosulphophthalein (BSP) for hOATP1B3. These substrates were incubated either alone or in the presence of iohexol (2 or 20 mM) or the respective positive control inhibitor (5 μM cyclosporine A for hOATP1B1 and hOATP1B3). Incubation times were 1 minute for hOATP1B1 and 7 minutes for hOATP1B3. The accumulation of radio-labelled probe substrates (^3^H) in the cell lysate is determined using a Tri-Carb 2810 liquid scintillation counter (PerkinElmer, Waltham, MA, USA) as described previously.[^1^](#_ENREF_1) Cellular protein amount was determined in parallel from 6 paired wells per cell line and experimental day, using the Bradford method.[^2^](#_ENREF_2) The 1X lysis buffer (5X lysis buffer from Promega, diluted 1+4 [v/v] in ddH_2_O) was used as the lysis buffer. Protein was measured using 1X Bradford reagent (5X stock reagent from Carl Roth, Karlsruhe, Germany, diluted 1+4 [v/v] in ddH₂O) and a Berthold Technologies TriStar2 LB942 spectrophotometer (Bad Wildbad, Germany) with bovine serum albumin as the standard.

### Uptake assay for hMDR1

Inside-out membrane vesicles (1.5 mg/mL) from HEK293 cells, including both hMDR1-vesicles and control vesicles, were purchased from Pharm Tox, Netherlands. These vesicles were used to measure the ATP-dependent uptake of a radiolabeled probe substrate using the rapid filtration technique. The uptake was initiated by rapidly mixing the membrane suspension (7.5 µg protein) with the pre-warmed (37°C) vesicle buffer containing the radio-labeled substrate (0.01 µM ^3^H-NMQ [N-methyl-quinidine]) either alone or in combination with iohexol (2 mM or 20 mM) or the respective positive control inhibitor (5 μM Elacridar) as well as 4 mM ATP and 10 mM MgCl_2_. After a 1 min incubation, the uptake was terminated by the addition of 500 µL of ice-cold Phosphate Buffered Saline (PBS) buffer. The mixture was immediately filtered through a glass fiber filter (Whatman GF/C) under vacuum. The filter was washed with ice-cold PBS buffer and dried under vacuum, before transferring the filter into a 6 mL scintillation vial. The measurement of the radiolabeled content was performed using the same technique employed in the uptake assays for hOATP1B1 and hOATP1B3.

### Data analysis

R version 4.2.1 (R Foundation for Statistical Computing, Vienna, Austria) was used for statistical evaluations and data visualization. The data analysis approach for the *in vitro* study was detailed by Hsin CH *et al.*.[^1^](#_ENREF_1) In brief, for transport assays, the initial uptake rate was calculated by normalizing the substrate concentration to both the protein concentration and the incubation time for each dish or well. The transporter-mediated uptake rate (net uptake) was determined by performing an element-wise subtraction of uptake rates from different conditions. First, all possible pairs of uptake rates were formed, with each pair consisting of one uptake rate from cells expressing the transporter or vesicles containing the transporter, and one uptake rate from control cells (vector-only or without transporter expression) or control vesicles within the same assay. Net uptake rates were determined by subtracting the uptake rate of the control cells or vesicles from that of the transporter-expressing cells or vesicles for each pair. The overall net uptake for a given transporter was then determined as the median, along with the 95% confidence intervals (CIs), of the net uptake rates across all assays.

## Part 2. *In vivo* study

### Participants and assessment

Twelve healthy volunteers, aged 18-60 years with a body mass index (BMI) between 18.5 and 30 kg/m², were enrolled in the relevant part of the study once all screening results were available and the subjects were considered eligible. The screening process included a review of medical history, a physical examination, a 12-lead standard ECG, routine laboratory tests, serological blood tests for hepatitis B, hepatitis C, and HIV, a urine analysis for substances of abuse, an alcohol breath test, measurement of thyroid-stimulating hormone serum levels, and, for female volunteers, a pregnancy test. The main exclusion criteria included contraindications to iohexol, significant clinical or laboratory abnormalities, current medication use, smoking, drug addiction, pregnancy, or breastfeeding.

Adverse events were monitored at multiple time points: within 1 hour before the administration of iohexol, and then at 10 minutes, 30 minutes, 1 hour, and 2 hours post-dose, as well as at the end of hospitalization for each period and during the end-of-study evaluation. Vital signs were recorded at the same time points as adverse events. Overall health was reassessed at the end of the study.

### Treatments

Participants will be randomly assigned to one of six sequences (1-2-3, 2-3-1, 3-1-2, 3-2-1, 1-3-2, 2-1-3) with each sequence comprising three treatments administered on separate occasions:

1) a 259 mg dose of iohexol under fasting conditions (“test”),

2) a 3235 mg dose of iohexol under fasting conditions (“reference”),

3) a 3235 mg dose of iohexol with the ingestion of boiled beef (not reported here).

For each treatment, either 5 ml (containing 3235 mg) or 0.4 ml (containing 259 mg) of iohexol solution (ACCUPAQUE™ 300 Injektionslösung)[^3^](#_ENREF_3) was injected intravenously as a single bolus over 1-2 minutes or 0.1-0.2 minutes, respectively. The syringes containing iohexol were weighed before and after injection to ensure accurate dosing. During the "fasting conditions", participants were hospitalized from 2 hours before to 24 hours after the administration of iohexol.

### Non-compartmental analysis

#### Evaluation of pharmacokinetic parameters

Plasma pharmacokinetic (PK) parameters were calculated, including the terminal elimination rate constant (λ_z_), terminal plasma elimination half-life (t_1/2, λz_), the volume of distribution (V_z_),[^4^](#_ENREF_4) the area under the plasma concentration-time curve (AUC), which includes AUC from time zero to the last time point (AUC_0-t_) and AUC from time zero extrapolated to infinity (AUC_0-∞_), and plasma clearance (CL). The λz value was determined by linear regression analysis of the terminal log-linear phase of individual concentration-time curves, using the last three concentration values above the lower limit of quantification (LLOQ). This approach ensures an accurate reflection of the terminal slope. The t_1/2, λz_ was calculated as ln(2)/λ_z_, and the V_z_ was calculated as Dose/(λ_z_*AUC_0-∞_). AUC was determined using the linear log trapezoidal rule. AUC_0-∞_ was calculated as AUC_0–t_ + C_last_/λ_z_, where the C_last_ is the concentration of the last time point with measurable concentration. CL was calculated as Dose/AUC_0-∞_. Urine iohexol PK parameters were also calculated, including the cumulative urinary excretion of unchanged iohexol from administration to the last time point (Ae_0-t_), the maximum observed excretion rate (R_max_), and the percentage of the administered iohexol dose recovered in urine, referred to as urinary recovery.

#### Statistical methods

Statistical evaluations for non-compartmental analysis (NCA) and bioequivalence analysis were performed using PKanalix 2024R1 (Lixoft SAS, a Simulations Plus company, Paris, France). CL estimates and urinary recovery, derived from NCA, were compared between the reference and test doses using the standard bioequivalence approach.[^5^](#_ENREF_5) Log-transformed values of CL and urinary recovery were evaluated using an analysis of variance model with fixed effects for sequence, subject within sequence, period, and dose (instead of “preparation” for bioequivalence assessment). The point estimate and 90% confidence interval (CI) for the test-to-reference ratio of CL and urinary recovery were calculated. The null hypothesis of “a relevant difference in CL between doses” was rejected, and CL was considered to be dose-linear if the 90 % CI was completely within the standard bioequivalence boundaries of 80–125%. The sample size calculation assumed that the intra-individual coefficient of variation (CV_intra_) for GFR measurement with iohexol would not exceed 11.4% in the general population.[^6^](#_ENREF_6) With this assumed CV_intra_ and a true ratio of 0.95 ≤ µ_test_/µ_reference_ ≤ 1.05, a sample size of 8 subjects would be sufficient to reject the null hypothesis of a significant difference in CL between doses, with a two-sided alpha of 0.05 and a power of at least 80%. However, since a minimum of 12 subjects is required for a bioequivalence study, we included 12 subjects.

### Population pharmacokinetic analysis

#### Assessing the impact of implausible data

We identified slight leakage during the injection of the test iohexol dose in one subject. To evaluate its impact on the final parameter estimates, we used two approaches: (i) conducting a sensitivity analysis by comparing parameter estimates across three models—the final model, a model excluding early post-dose plasma samples from the subject, and a model excluding all data from this period for the subject. (ii) expanding the model to describe absorption from an additional para-venous compartment for the administration of iohexol in this subject and period only.

## LC-MS/MS analysis

For quantification, all drugs except for PAH were measured using an API 5000 device (AB Sciex Germany GmbH, Darmstadt, Germany), connected to an Agilent 1200 HPLC binary pump and an Agilent 1260 Infinity autosampler (Agilent Technologies Deutschland GmbH, Waldbronn, Germany). The concentrations of PAH in cell lysis samples were determined using an API 4000 QTRAP device (AB Sciex Germany GmbH, Darmstadt, Germany), connected to a Shimadzu LC-20AD HPLC pump and a Shimadzu SIL-20AC HT autosampler (Shimadzu Deutschland GmbH, Duisburg, Germany). For all measurements, the column temperature was maintained at 40°C, and the samples were stored at 4°C in an autosampler until injection. Instrument control and data acquisition were performed using Analyst 1.6.2 software (AB Sciex Germany GmbH, Darmstadt, Germany). Quantification was based on peak area, with calibration functions determined using weighted least-squares linear regression. Specific quantitation methods are detailed below, and the liquid chromatography conditions and tandem mass spectrometry parameters are provided in Tables S1 and S2, respectively.

### Quantitation of iohexol in plasma and urine

For the preparation of plasma and urine samples, 50 µL of each plasma sample or 10 µL of each urine sample was dispensed into a 96-well plate. To each sample, 10 µL of an iohexol-d5 (internal standard) working solution (5 µg/mL) was added. Plasma samples were deproteinized using 200 µL of acetonitrile, while urine samples were deproteinized with 400 µL of acetonitrile. After vertexing the mixture for 30 seconds, it was centrifuged at room temperature for 15 minutes at 3000 × g. A 150-µL aliquot of the resulting supernatant was transferred to a 96-well plate for LC-MS/MS analysis. All assays for iohexol concentration in plasma and urine met the bioanalytical method validation criteria established by the US Food and Drug Administration (FDA)[^7^](#_ENREF_7) and the European Medicines Agency (EMA)[^8^](#_ENREF_8). The LLOQs were 25 ng/mL for plasma and 125 ng/mL for urine. Accuracy for plasma and urine quality control (QC) samples, including LLOQ, low, medium, and high concentrations, ranged from 89.07% to 109.24% of the nominal values. Intra-day and inter-day precision, expressed as coefficients of variation, were below 6.08% for plasma samples and 6.76% for urine samples.

### Quantitation of probe drugs in cell lysate

For sample preparation, cell lysate samples from uptake experiments were vortexed and centrifuged at room temperature for 2 minutes at 16,100 × g. A 150-µL aliquot of the resulting supernatant was then transferred to a 96-well plate for LC-MS/MS analysis.

# RESULTS

## Part 2 *In vivo* study

### Dataset

#### 1. Missing data

Urinary excretion data were unavailable for the 2–4 hour interval in Subject 8 following the reference dose and for the 14–16 hour interval in Subject 9 following the test dose (see Figure S4). Consequently, data from these two subjects were excluded from the NCA.

#### 2. Implausible data

Figure S4 shows that the initial plasma concentrations of iohexol in Subject 6 after the test dose exhibited an absorption-like behavior, suggesting that at least part of the dose might have been administered paravenously.

### Safety

In total, 7 of 12 subjects reported 9 adverse events. None of these were related to iohexol, while one rated as severe (syncope after venipuncture prior to scheduled iohexol administration) was associated with study procedures. Four subjects used single doses of concomitant medications for the adverse events, including ibuprofen, paracetamol, and pantoprazole. Ibuprofen, as the only one of these drugs with a relevant effect on renal function, was taken between study periods and is therefore not expected to have influenced the results.

### Population pharmacokinetic analysis

#### Model evaluation

The visual predictive check (VPC) results (Figure S5) indicated that the medians and 10^th^ and 90^th^ percentiles of the simulated data from the final model were in acceptable agreement with the observed data. The goodness-of-fit (GOF) plots (Figure S6) demonstrated that the final model accurately described the observed iohexol plasma concentrations and urinary excretion. There was good agreement between the observed and individual/population-predicted plasma concentrations and urinary amounts. Conditional weighted residuals (CWRES) were randomly scattered around zero, suggesting no systematic deviations in the model. However, several outliers in plasma concentrations during the early phase after administration of the test dose were observed in the GOF plots (Figure S6 (b)), primarily from Subject 6, who displayed an apparent absorption phase, as previously described. The final point estimates and bootstrap statistics of PK parameters are summarized in Table 3, with no indications of overparameterization in any of the model diagnostics or the bootstrap results.

#### Assessing the impact of implausible data

The sensitivity analysis of parameter estimates from models using different datasets is presented in Table S5. The parameter estimates remained nearly unchanged across models developed using the entire dataset, the dataset excluding plasma samples taken within 45 minutes after the test dose for the subject with delayed absorption, and the dataset excluding the respective period. Modeling an absorption phase for this subject and period resulted in a first-order absorption rate constant of 0.876 h^-1^, a relative bioavailability of 93.6%, and an improvement in OFV by 185 points, while other parameters were almost identical (not shown).

# SUPPLEMENTARY TABLES

## Table S1 Liquid chromatography condition for analytes.

| **Analyte**  **(IS)** | **Matrix** | **Standard curve range** | **Injection**  **volume (μL)** | **Flow rate (mL/min)** | **Gradient elution**  **%B^a^ (min)** | **Columns** |
| --- | --- | --- | --- | --- | --- | --- |
| Iohexol  (Iohexol-d5) | Plasma | 25 – 2500 ng/mL | 10 | 0.3 | 90 (0.0) → 90 (0.2) → 15(1.0) → 15(3.5) → 90 (4.0) → 90 (6.0) | SeQuant^®^ ZIC^®^-HILIC column  (2.1 x 100 mm, 5 μm, 200Å, Merck KGaA, Darmstadt, Germany) |
|  | Urine | 0.125 – 12.5 μg/mL |  |  |  |  |
|  | Cell lysate | 0.5 – 32 ng/mL |  |  |  |  |
| metformin | Cell lysate | 3.125 – 800 ng/mL  (for hOCT1) | 5 | 0.3 | 85 (0.0) → 85 (0.1) → 15(0.5) → 15(3.5) → 85 (4.0) → 85 (7.0) | SeQuant^®^ ZIC^®^-HILIC column  (2.1 x 100 mm, 5 μm, 200Å, Merck KGaA, Darmstadt, Germany) |
|  |  | 0.195 – 100 ng/mL  (for hOCT2) |  |  |  |  |
| PAH | Cell lysate | 0.49 – 1000 ng/mL | 20 | 0.4 | 80 (0.0) → 80 (0.25) → 20(2.0) → 20(3.0) → 80 (4.0) → 80 (5.0) | Waters Atlantis HILIC Silica Column 3.0 x 50 mm 5 μm, combined with Altantis^®^HILIC, 5μM VanGuard^®^ Cartridge  (Waters Chromatography Ireland Limited, Dublin, Ireland) |
| E3S | Cell lysate | 0.78 – 100 ng/mL | 10 | 0.2 | 35 (0.0) → 35 (0.25) → 90(3.0) → 90(3.5) → 35 (5.5) → 35 (9.0) | Xbridge^®^Shield RP18 3.5um, 3.0x100mm Column, combined with Xbridge^®^BEH Shield RP18 3.5μM, VanGuard^®^ Cartridge  (Waters Chromatography Ireland Limited, Dublin, Ireland) |
| MPP+ | Cell lysate | 0.39 – 25 ng/mL | 5 | 0.3 | 90 (0.0) → 90 (0.1) → 15(1.0) → 15(3.5) → 90 (4.5) → 90 (6.0) | SeQuant^®^ ZIC^®^-HILIC column  (2.1 x 100 mm, 5 μm, 200Å, Merck KGaA, Darmstadt, Germany) |

IS, internal standard; PAH, para-amino hippuric acid; E3S, Estrone-3-Sulfate; MPP+, 1-Methyl-4-phenylpyridinium.

^a^ The gradient solvent systems for the compounds are as follows: Iohexol (Iohexol-d5) and MPP+ were analyzed with 0.1% formic acid in water (A) and acetonitrile with 0.1% formic acid (B); Metformin was assessed with 10 mM ammonium formate (pH 3.75) (A) and acetonitrile (B); PAH was determined using 10 mM ammonium acetate (pH 4) (A) and methanol with 0.1% formic acid (B); and E3S was evaluated using 10 mM ammonium acetate (pH 8.9) (A) and methanol (B).

## Table S2 Optimized tandem mass spectrometry parameters for analytes.

| **Analyte** | **Mode** | **Ion transition (Da)** | **DP (volts)** | **CE (volts)** | **CXP (volts)** |
| --- | --- | --- | --- | --- | --- |
| iohexol | positive | 821.937 → 374.700 | 196.000 | 65.000 | 16.000 |
| Iohexol-d_5_  (IS) | positive | 826.911 → 607.900 | 146.000 | 39.000 | 28.000 |
| PAH | positive | 195.134 → 120.100 | 36.000 | 15.000 | 2.000 |
| E3S | negative | 349.142 → 269.000 | -170.000 | -48.000 | -27.000 |
| MPP+ | positive | 170.123 → 128.200 | 131.000 | 45.000 | 26.000 |
| metformin | positive | 130.176 → 60.000 | 81.000 | 19.000 | 14.000 |

DP, declustering potential; CE, collision energy; CXP, collision cell exit potential; IS, internal standard; PAH, para-amino hippuric acid; E3S, Estrone-3-Sulfate; MPP+, 1-Methyl-4-phenylpyridinium.

## Table S3 Evaluation of *in vitro* system quality and inhibitory effects of iohexol on these systems.

| **Transporter** | ***In vitro* systems** | **Inhibitory effect (% reduction of specific activity)^b^** | | | |
| --- | --- | --- | --- | --- | --- |
|  | **Expression on vs. off^a^** | **Prototypical inhibitors** | **1 mM or 2 mM iohexol** | **20 mM iohexol** |  |
| hOAT1 | 250 (240 to 260) | 70 (70 to 71) | -0.45 (-0.53 to 1.3) | 3.1 (2.8 to 4.3) |  |
| hOAT3 | 13 (11 to 16) | 94 (94 to 94) | -4 (-5.6 to -2.4) | -3.5 (-7.5 to -0.29) |  |
| hOCT1 | 17 (16 to 20) | 90 (89 to 90) | -9.8 (-12 to -8) | -22 (-33 to -5.4) |  |
| hOCT2 | 19 (15 to 27) | 49 (48 to 50) | -13 (-14 to -10) | -23 (-26 to -22) |  |
| hMATE1 | 18 (17 to 20) | 95 (95 to 95) | 5.4 (4.5 to 6.4) | 12 (11 to 14) |  |
| hMATE2K | 8 (7.3 to 8.8) | 75 (75 to 76) | 14 (13 to 16) | 26 (25 to 28) |  |
| hOATP1B1 | 17 (16 to 20) | 83 (82 to 83) | -6.9 (-9.2 to -4.5) | 5.5 (4.6 to 7.6) |  |
| hOATP1B3 | 3.3 (3.3 to 3.8) | 94 (90 to 96) | -2.5 (-3.8 to -1.6) | -21 (-24 to -18) |  |
| hMDR1 | 5.4 (4.8 to 5.9) | 99 (97 to 100) | -4.9 (-10 to -1.2) | 21 (17 to 24) |  |

Stably transfected 293 cells, either with (expression on; n = 4 for hOAT3, n = 3 for other transporters) or without (expression off; n = 4 for hOAT3, n = 3 for other transporters) expression of hOAT1, hOAT3, hOCT1, hOCT2, hMATE1, or hMATE2K, were incubated with transporter-specific probe substrates alone, with prototypical inhibitors, or with 1 mM or 20 mM iohexol. Similarly, 293 cells stably expressing hOATP1B1, hOATP1B3, or hMDR1 vesicles (transporter-expressing; n = 3), and corresponding controls (empty-vector transfected cells or control vesicles; n = 3), were incubated with probe substrates alone, with prototypical inhibitors, or with 2 mM or 20 mM iohexol. Data are presented as the median values with 95% CIs across all experiments.

^a^ Uptake rate ratios of probe substrates in transporter-expressing cells or vesicles were normalized to uptake rates in control cells or vesicles through element-wise division.

^b^ Inhibitory effects of prototypical inhibitors and iohexol were calculated as the percentage reduction in transporter activity relative to activity with the probe substrate alone, using element-wise division within each experiment.

## Table S4 Iohexol accumulation and uptake ratios in transporter-expressing and non-expressing 293 cells.

| **Transporter** | **Incubation time (minutes)** | **Differences in** **intracellular accumulation^a^**  **(pmol/mg protein)** | **Uptake ratio^b^** |
| --- | --- | --- | --- |
| hOAT1 | 10 | -0.39 (-3.4 to 1.1) | 0.91 (0.75 to 1.2) |
|  | 30 | 0.98 (-0.62 to 2.1) | 1.1 (0.95 to 1.3) |
| hOAT3 | 10 | 0.6 (0.1 to 2) | 1.1 (1 to 1.4) |
|  | 30 | 0.89 (0.19 to 1.5) | 1.2 (1.1 to 1.3) |
| hOCT1 | 10 | 0.16 (-1.8 to 0.79) | 1 (0.84 to 1.1) |
|  | 30 | -0.51 (-1.1 to -0.15) | 0.89 (0.79 to 0.97) |
| hOCT2 | 10 | 1.3 (0.007 to 2.6) | 1.1 (1 to 1.2) |
|  | 30 | 1.5 (-1.4 to 2.9) | 1.1 (0.9 to 1.3) |
| hMATE1 | 10 | 0.36 (-1.2 to 1.7) | 1.1 (0.84 to 1.2) |
|  | 30 | -0.67 (-1.6 to 0.61) | 0.92 (0.78 to 1.1) |
| hMATE2K | 10 | -0.36 (-1.1 to 0.24) | 0.95 (0.81 to 1.1) |
|  | 30 | 0.62 (-0.38 to 1.5) | 1.1 (0.93 to 1.4) |

Stably transfected 293 cells, either expressing (n = 4 for hOAT3; n = 3 for other transporters) or not expressing (n = 4 for hOAT3; n = 3 for other transporters) hOAT1, hOAT3, hOCT1, hOCT2, hMATE1, or hMATE2K, were incubated with 10 μM iohexol for 10 and 30 minutes, respectively. Each experiment was performed in triplicate, with each assay conducted on a separate day. Data are presented as the median values with 95% CIs across all experiments.

^a^ The difference in iohexol accumulation between transporter-expressing and control cells was determined by element-wise subtraction of intracellular iohexol accumulations under "expression off" from that under "expression on" conditions for each transporter and experimental group.

^b^ The ratio of iohexol uptake rates in transporter-expressing cells compared to non-expressing cells was calculated through element-wise division within each transporter and experimental group.

## Table S5 Sensitivity analysis of parameter estimates from models using different datasets.

|  | Final model^a^ | | | Model 1^b^ | | | | Model 2^c^ | |  |
| --- | --- | --- | --- | --- | --- | --- | --- | --- | --- | --- |
| Parameters | | Estimate | RSE (%) | | Estimate | RSE (%) | Estimate | | RSE (%) | |
| CL_R_ (L/h) | | 5.50 | 4.22 | | 5.45 | 4.33 | 5.49 | | 4.22 | |
| V_1_ (L) | | 9.06 | 4.88 | | 8.91 | 4.49 | 8.90 | | 4.64 | |
| Q_1_ (L/h) | | 0.221 | 14.7 | | 0.219 | 15.3 | 0.227 | | 16.2 | |
| V_2_ (L) | | 1.56 | 7.74 | | 1.54 | 8.31 | 1.57 | | 8.24 | |
| Q_2_ (L/h) | | 5.84 | 18.5 | | 5.53 | 17.6 | 5.38 | | 17.7 | |
| V_3_ (L) | | 4.34 | 7.37 | | 4.30 | 6.88 | 4.30 | | 6.65 | |
| Inter-individual variability (CV%) | | | | | | | | | |  |
| CL_R_ | | 14.0 | 29.5 | | 14.4 | 28.9 | 14.2 | | 28.5 | |
| V_1_ | | 16.6 | 24.0 | | 16.8 | 25.4 | 17.2 | | 21.5 | |
| V_2_ | | 14.0 | 21.3 | | 15.1 | 20.1 | 13.8 | | 22.9 | |
| V_3_ | | 15.5 | 18.8 | | 15.0 | 17.9 | 13.5 | | 19.5 | |
| Inter-occasion variability (CV%) | | | | | | | | | |  |
| CL_R_ | | 2.51 | 29.1 | | 3.69 | 33.2 | 2.33 | | 26.3 | |
| V_1_ | | 6.23 | 39.2 | | 4.82 | 48.4 | 3.87 | | 36.2 | |
| Q_2_ | | 29.2 | 50.0 | | 23.7 | 40.6 | 17.1 | | 47.9 | |
| Residual variability (CV%) | | | | | | | | | |  |
| plasma | | 11.3 | 17.9 | | 8.94 | 4.40 | 8.84 | | 4.85 | |
| urine | | 25.0 | 20.7 | | 25.5 | 20.4 | 25.4 | | 21.0 | |

RSE%, relative standard error expressed as a percentage; CL_R_, renal clearance; Q_1_ and Q_2_, intercompartmental clearances; V_1_, central volume of distribution; V_2_ and V_3_ peripheral volumes of distribution; CV%, coefficient of variation expressed as a percentage, CV% for Inter-individual and Inter-occasion variability computed as$\sqrt{exp\left( \omega^{2} \right)-1}$, CV% for residual unexplained variability computed as$\sqrt{exp\left( \sigma^{2} \right)-1}$.

^a^ The final model was developed using the entire dataset.

^b^ Model 1 was developed excluding samples taken within 45 minutes after the test dose for the subject with delayed absorption

^c^ Model 2 was developed excluding all data from the subject with delayed absorption when receiving the iohexol test dose.

# SUPPLEMENTARY FIGURES


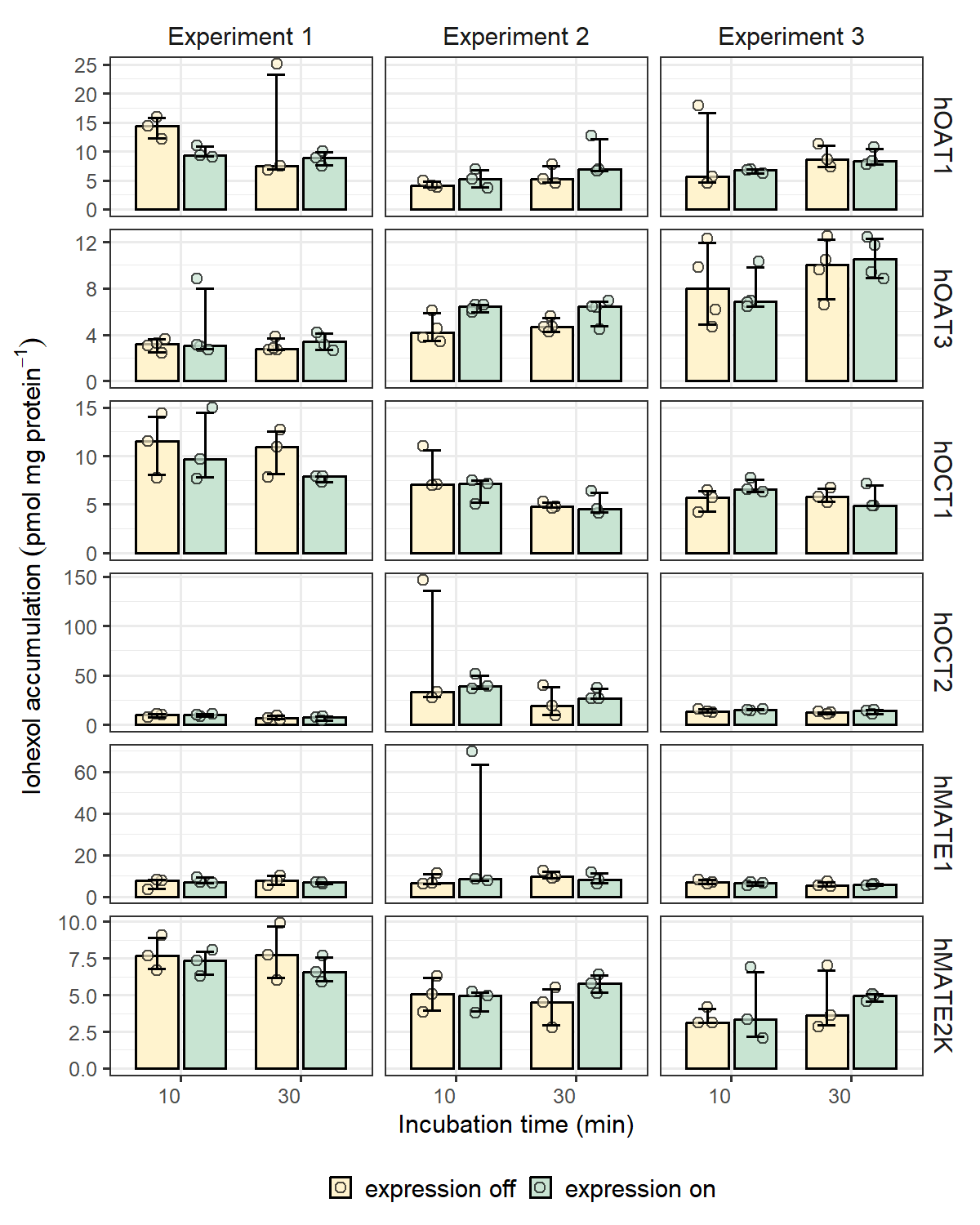


# Figure S1 The accumulation of iohexol in 293 cells with or without the expression of hOAT1, hOAT3, hOCT1, hOCT2, hMATE1, or hMATE2K.

Stably transfected 293 cells, either expressing (expression on; n=3 or 4) or not expressing (expression off; n=3 or 4) hOAT1, hOAT3, hOCT1, hOCT2, hMATE1, or hMATE2K, were incubated with 10 μM iohexol for 10 and 30 minutes, respectively. Each dot represents an individual dish. The columns and their error bars indicate the 5^th^, 50^th^, and 95^th^ percentiles of the data.


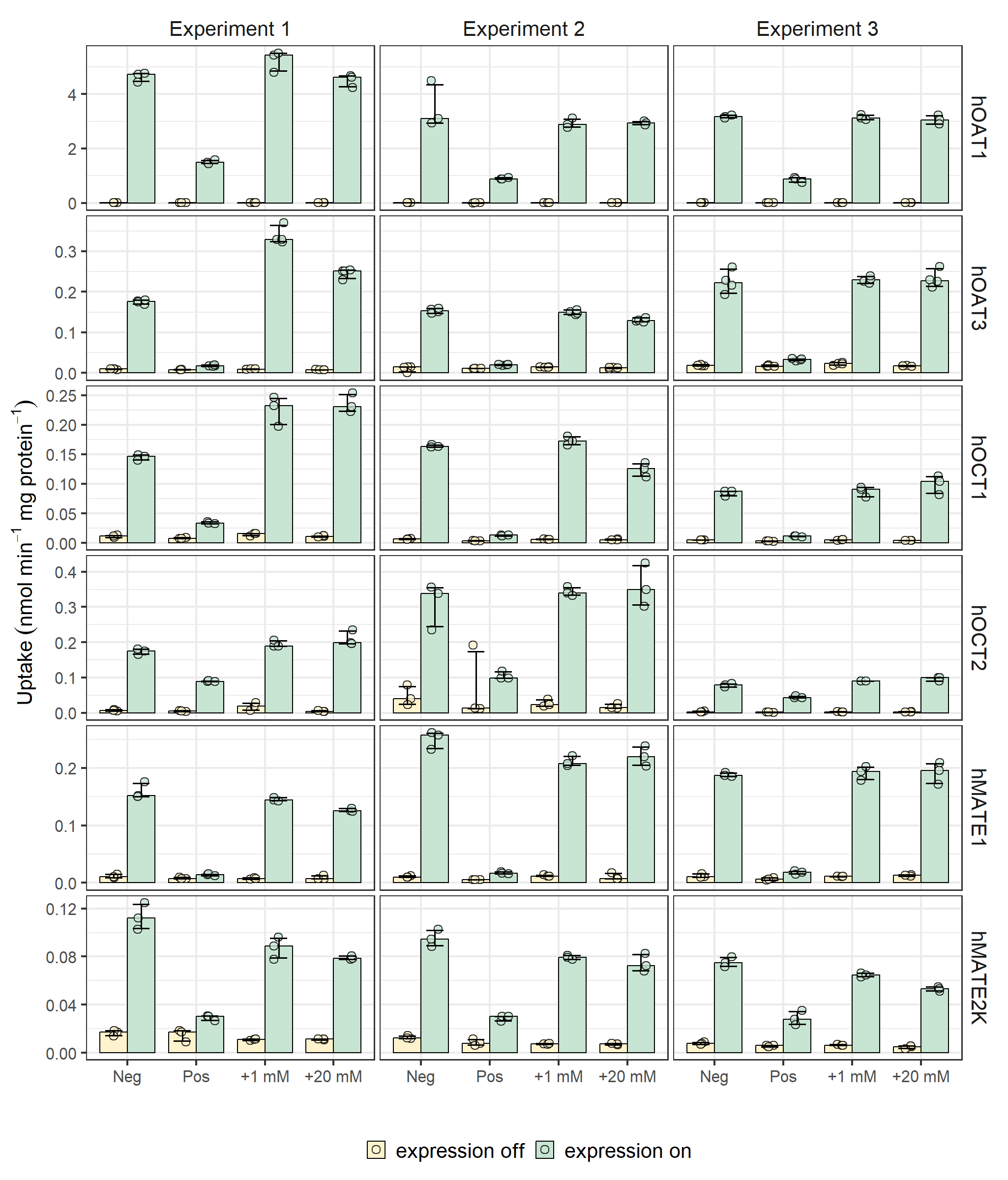


## Figure S2 Impact of iohexol on the transport of standard substrates mediated by hOAT1, hOAT3, hOCT1, hOCT2, hMATE1, or hMATE2K.

Stably transfected 293 cells, either with (expression on; n=3 or 4) or without (expression off; n=3 or 4) the expression of hOAT1, hOAT3, hOCT1, hOCT2, hMATE1, or hMATE2K were incubated with the probe substrate for each transporter. The conditions included incubation with the standard substrate alone (Neg), with the respective positive inhibitor (Pos), or with 1 mM iohexol (+1 mM) or 20 mM iohexol (+20 mM). Each experiment was conducted three times. Each dot represents an individual dish. The columns and their error bars indicate the 5^th^, 50^th^, and 95^th^ percentiles of the data.


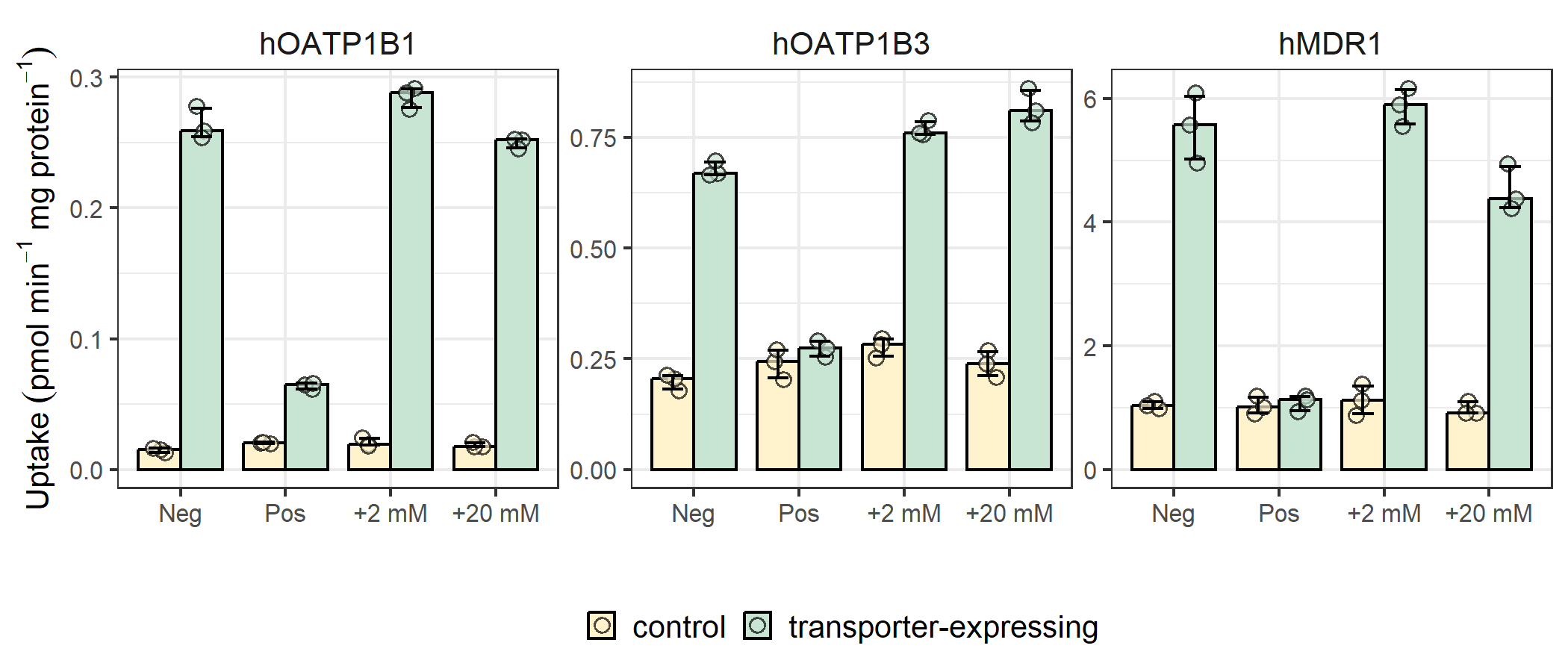


## Figure S3 Impact of iohexol on the transport of standard substrates mediated by hOATP1B1, hOATP1B3, or hMDR1.

Stably transfected 293 cells expressing hOATP1B1, hOATP1B3, or hMDR1-expressing vesicles (transporter-expressing; n=3), along with control cells transfected with empty vectors or control vesicles (control; n=3), were incubated with transporter-specific probe substrates. Incubation conditions included: probe substrate alone (Neg), with a positive control inhibitor (Pos), or with iohexol at 2 mM (+2 mM) or 20 mM (+20 mM). Each dot represents a single well. Columns and error bars represent the 5^th^, 50^th^, and 95^th^ percentiles.


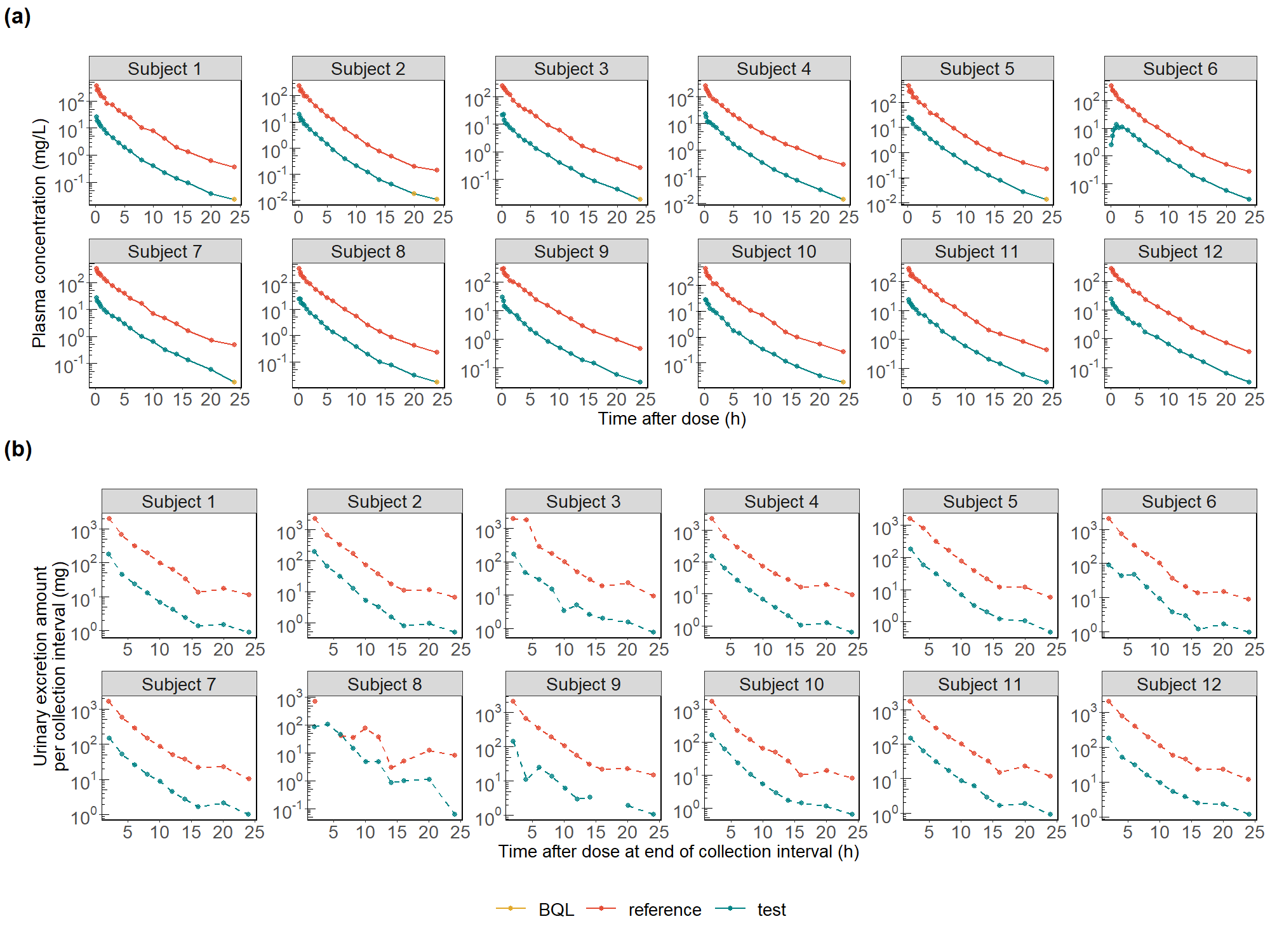


## Figure S4 Time-course of log-scaled iohexol plasma concentrations and urinary excretion.

(a) Log-scaled iohexol plasma concentrations over time following the reference and test doses, respectively. (b) Log-scaled urinary excretion amounts per collection interval following the reference and test doses, respectively. Data points are represented by dots: non-BQL (above the quantification limit) values are color-coded to match their respective lines, while BQL (below the quantification limit) values are highlighted in yellow. Note: urinary excretion data were unavailable for the 2–4 hour collection interval for Subject 8 after the reference dose, and for the 14–16 hour interval for Subject 9 after the test dose.


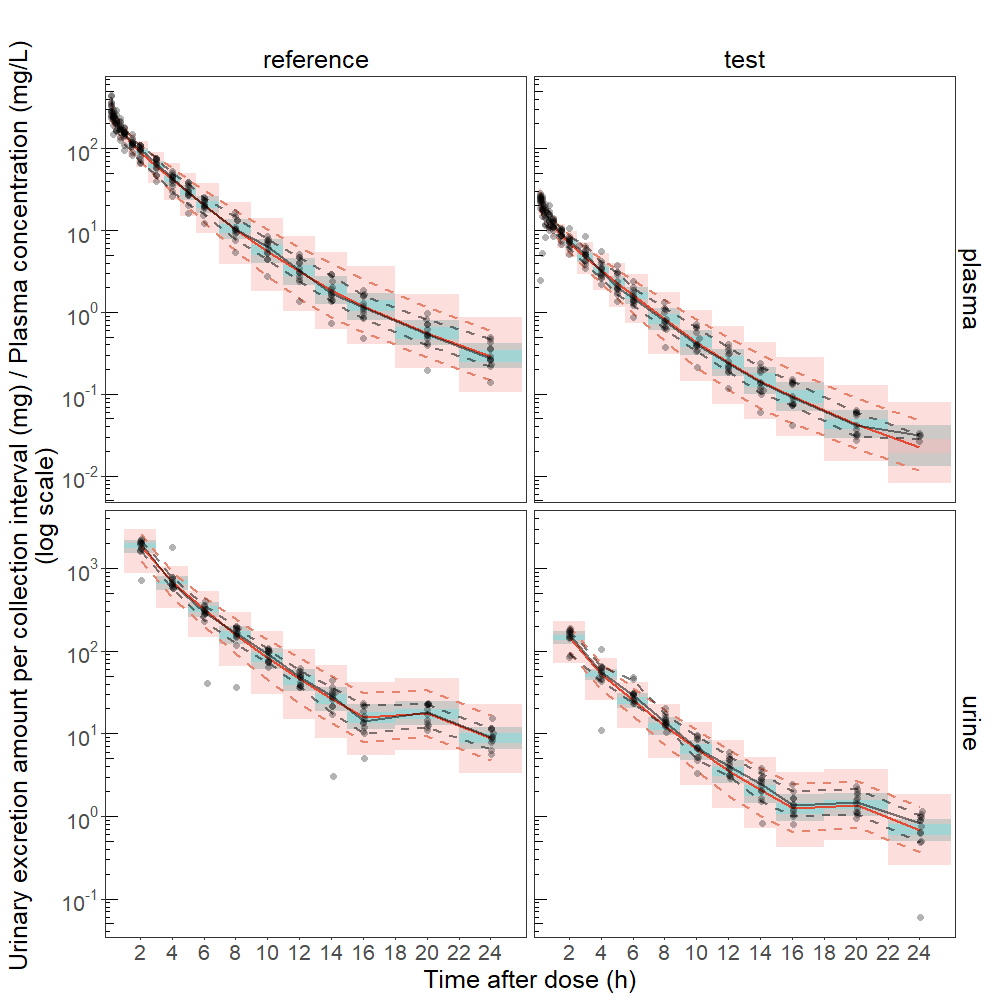


## Figure S5 Visual predictive check (n = 1000) for the final model stratified by plasma and urine data, and categorized by reference and test dose levels.

Dots illustrate observed data points. Solid (dashed) black and red lines represent medians (10^th^, and 90^th^ percentiles) of observations and simulated data, respectively; red, green, and red areas represent 95% confidence intervals of the 10^th^, 50^th^, and 90^th^ percentiles of simulated data. The X-axis represents the sampling time (hours) for plasma data and the time at the end of the collection interval (hours) for urine data.


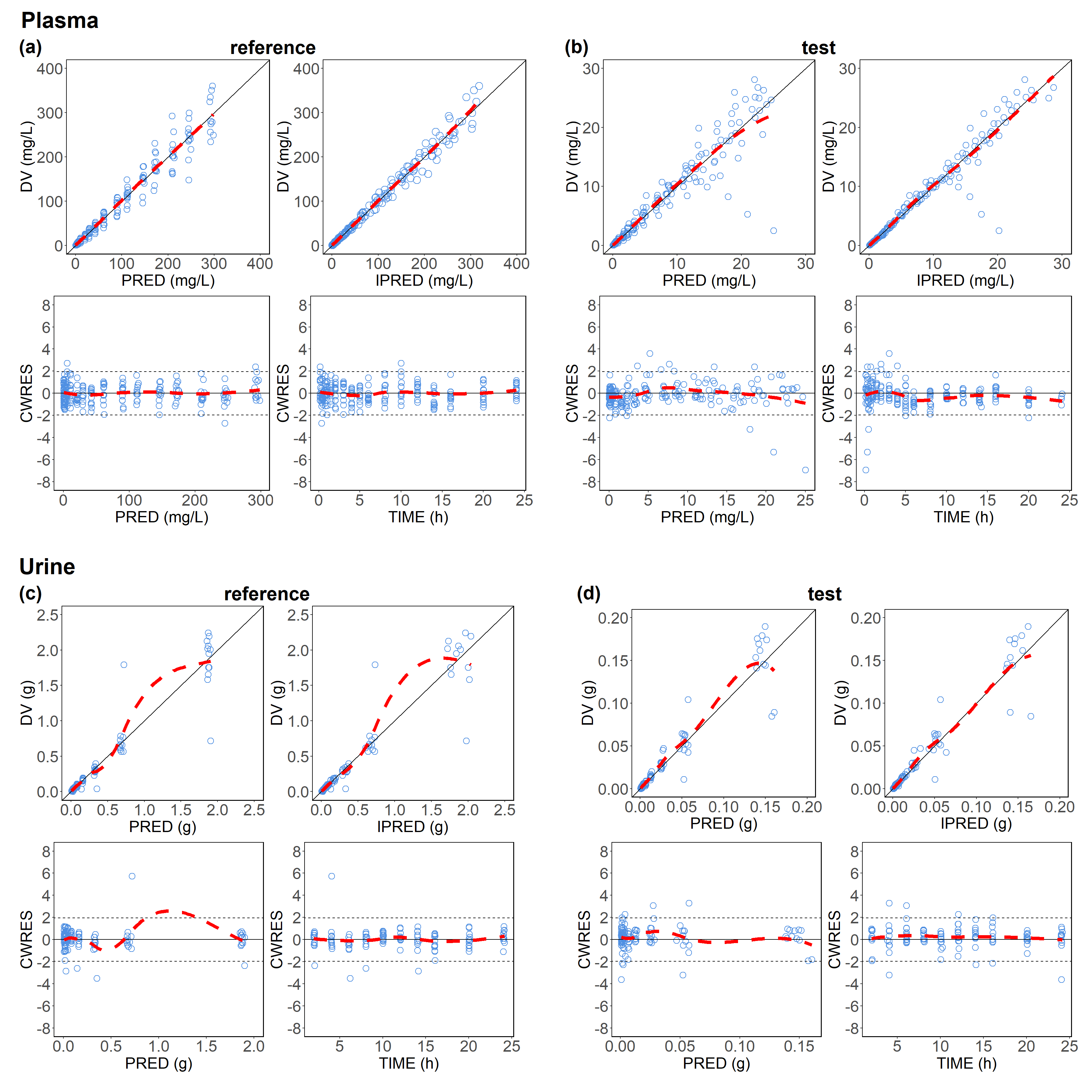


## Figure S6 Goodness-of-Fit plots for the final model.

Panels (a) and (b) show plasma concentrations following the reference and test doses, respectively. Panels (c) and (d) display urinary excretion amounts following the reference and test doses, respectively. DV (dependent variable) represents observed plasma concentrations or urinary excretion amounts per collection interval. PRED (population prediction) indicates the predicted plasma concentration or urinary excretion amount per collection interval for the population; IPRED (individual prediction) denotes the predicted plasma concentration or urinary excretion amount per collection interval for each individual. CWRES represents conditional weighted residuals; TIME represents plasma sampling times or the end of each urine collection interval. Data points are depicted as open circles. Solid black lines represent the line of identity or a residual of 0, while dashed black lines show reference lines at y = ±1.96. Red dashed lines indicate locally weighted smoothing lines.

# REFERENCES

1. Hsin CH, Kuehne A, Gu Y, et al. In vitro validation of an in vivo phenotyping drug cocktail for major drug transporters in humans. *Eur J Pharm Sci*. Jul 1 2023;186:106459. doi:10.1016/j.ejps.2023.106459

2. Bradford MM. A rapid and sensitive method for the quantitation of microgram quantities of protein utilizing the principle of protein-dye binding. *Anal Biochem*. May 7 1976;72:248-54. doi:10.1006/abio.1976.9999

3. GE Healthcare Buchler GmbH & Co. KG. Fachinformation ACCUPAQUE™ 240, - 300, - 350. Juni 2021;

4. Toutain PL, Bousquet-Mélou A. Volumes of distribution. *J Vet Pharmacol Ther*. Dec 2004;27(6):441-53. doi:10.1111/j.1365-2885.2004.00602.x

5. European Medicines Agency. (2010). Committee for Medicinal Products for Human Use (CHMP) Guideline on the Investigation of Bioequivalence. CPMP/EWP/QWP/1401/98 Rev 1/Corr. Available from: https://www.ema.europa.eu/en/documents/scientific-guideline/guideline-investigation-bioequivalence-rev1_en.pdf. Accessed May 16, 2024.

6. Delanaye P, Ebert N, Melsom T, et al. Iohexol plasma clearance for measuring glomerular filtration rate in clinical practice and research: a review. Part 1: How to measure glomerular filtration rate with iohexol? *Clinical kidney journal*. 2016;9(5):682-699.

7. U.S. Department of Health and Human Services; Food and Drug Administration; Center for Drug Evaluation and Research (CDER). (2018). Bioanalytical Method Validation: Guidance for Industry. Available from: https://www.fda.gov/files/drugs/published/Bioanalytical-Method-Validation-Guidance-for-Industry.pdf. Accessed July 30, 2024.

8. European Medicines Agency, Committee for Medicinal Products for Human Use. (2022). ICH Guideline M10 on Bioanalytical Method Validation and Study Sample Analysis. EMA/CHMP/ICH/172948/2019. Available from: https://www.ema.europa.eu/en/documents/scientific-guideline/ich-guideline-m10-bioanalytical-method-validation-step-5_en.pdf. Accessed July 30, 2024
